# Using meta‐analysis to understand the impacts of dietary protein and fat content on the composition of fecal microbiota of domestic dogs (*Canis lupus familiaris*): A pilot study

**DOI:** 10.1002/mbo3.1404

**Published:** 2024-03-21

**Authors:** Francis D. Phimister, Rachel C. Anderson, David G. Thomas, Michelle J. Farquhar, Paul Maclean, Ruy Jauregui, Wayne Young, Christina F. Butowski, Emma N. Bermingham

**Affiliations:** ^1^ AgResearch Ltd Manawatu‐Whanganui New Zealand; ^2^ School of Agricultural and Environment Massey University Manawatu‐Whanganui New Zealand; ^3^ Waltham Petcare Science Institute Waltham on the Wolds Leicestershire UK

**Keywords:** canine, crude fat, crude protein, microbiome

## Abstract

The interplay between diet and fecal microbiota composition is garnering increased interest across various host species, including domestic dogs. While the influence of dietary macronutrients and their associated microbial communities have been extensively reviewed, these reviews are descriptive and do not account for differences in microbial community analysis, nor do they standardize macronutrient content across studies. To address this, a meta‐analysis was performed to assess the impact of dietary crude protein (“protein”) and dietary crude fat (“fat”) on the fecal microbiota composition in healthy dogs. Sixteen publications met the eligibility criteria for the meta‐analysis, yielding a final data set of 314 dogs. Diets were classed as low, moderate, high, or supra in terms of protein or fat content. Sequence data from each publication were retrieved from public databases and reanalyzed using consistent bioinformatic pipelines. Analysis of community diversity indices and unsupervised clustering of the data with principal coordinate analysis revealed a small effect size and complete overlap between protein and fat levels at the overall community level. Supervised clustering through random forest analysis and partial least squares‐discriminant analysis indicated alterations in the fecal microbiota composition at a more individual taxonomic level, corresponding to the levels of protein or fat. The *Prevotellaceae Ga6A1 group* and *Enterococcus* were associated with increasing levels of protein, while *Allobaculum* and *Clostridium sensu stricto 13* were associated with increasing levels of fat. Interestingly, the random forest analyses revealed that *Sharpea*, despite its low relative abundance in the dog's fecal microbiome, was primarily responsible for the separation of the microbiome for both protein and fat. Future research should focus on validating and understanding the functional roles of these relatively low‐abundant genera.

## Graphical abstract

This meta‐analysis investigates the impact of dietary crude protein and fat on the fecal microbiota composition in healthy dogs. While overall community changes were minimal, individual taxonomic alterations were observed with increasing levels of protein and fat. The study highlights the role of low‐abundant genera like *Sharpea* in differentiating the microbiome based on dietary intake, suggesting a need for further research into their functional roles.

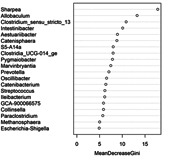

## 1 INTRODUCTION

The microbiota present in the gastrointestinal tract (GIT) of the domestic dog are a diverse and complex community of microorganisms (Moon et al., 2018; Valdes et al., 2018). The commensal bacteria observed in the GIT of the dog fall into one of the five most abundant bacterial phyla: *Bacteroidetes*, *Fusobacteria*, *Firmicutes*, *Proteobacteria*, and *Actinobacteria* (Honneffer et al., 2017). Generally, these communities are reflective of the environment, substrate availability, and functions of the area of the GIT they inhabit (Honneffer et al., 2017; Pilla & Suchodolski, 2020; Suchodolski et al., 2008). Obligate anaerobic bacteria, for example, are found predominantly in the anaerobic large intestine and belong predominantly to *Firmicutes* and/or are capable of fermenting dietary fibers (Honneffer et al., 2017; Panasevich et al., 2015; Suchodolski et al., 2008). In comparison, the oxygenated small intestine houses aerobic and facultative anaerobes and protein‐metabolizing bacteria, which commonly belong to *Proteobacteria* (Honneffer et al., 2017; Moon et al., 2018; Suchodolski et al., 2008). In general, a prevalence of *Fusobacteria* is associated with protein‐rich diets (Menke et al., 2014; Nelson et al., 2013; Zhu et al., 2018), an increased relative abundance of *Proteobacteria* is associated with protein metabolism (Moon et al., 2018), and increased levels of *Actinobacteria* can be seen with higher levels of dietary fats (Bermingham et al., 2017).

As with other species, changes to the diet of the dog can result in rapid shifts in the composition of fecal microbiota, which is used as a proxy for the gut microbiota (Allaway et al., 2020). As indicated by recent reviews in this research area, a large variety of dietary formats and/or dietary protein and fat sources have been investigated. These include bone and raw food, commercial extruded kibble diets (Alessandri et al., 2019; Beloshapka, 2013; Bermingham et al., 2017; Kim et al., 2017; Sandri et al., 2017, 2019, 2020; Schmidt et al., 2018), insects (Jarett et al., 2019), and nonanimal protein sources (Bresciani et al., 2018; Kerr et al., 2013; Reilly et al., 2021). Additionally, the impacts of specific ingredients such as dietary crude protein (referred to hereafter as protein) (Bermingham et al., 2017; Bermingham et al., unpublished; Bermudez Sanchez et al., 2020; Ephraim et al., 2020; Hang et al., 2012; Herstad et al., 2017; Li et al., 2017; Pinna et al., 2018), dietary crude fat/ether extract (referred to hereafter as fat) (Bermingham et al., unpublished; Herstad et al., 2017; Moinard et al., 2020; Schauf et al., 2018; Schmidt et al., 2018), carbohydrate (CHO) (Hang et al., 2012; Li et al., 2017; Schauf et al., 2018), and dietary fiber (Bermudez Sanchez et al., 2020; Biagi et al., 2010; Jackson & Jewell, 2016, 2019; Kerr et al., 2013; Nogueira et al., 2019; Panasevich et al., 2015; Sandri et al., 2020) have been assessed. However, there are inconsistencies between how diets are classified, which makes interpretation of the results challenging. For example, two separate studies both classified diets as “raw meat based” (Bermingham et al., 2017; Sandri et al., 2020); yet, one was almost completely CHO free (0.6% content by dry matter; % DM) (Bermingham et al., 2017), while the other contained 42%–43% DM CHO (Sandri et al., 2020). This suggests that a systematic review and/or meta‐analysis of the impacts of the dietary macronutrient content on the fecal microbiota in the dog may provide insights into the interactions between the host, diet, and microbiota.

Meta‐analyses are a statistical tool used to examine the results of multiple studies to minimize the bias that can be introduced in separate, smaller studies, while also allowing for the possibility of challenging a larger overall data set with new variables (Phillips, 2005). Thus, meta‐analyses are being used increasingly across medical and nutritional research (Haidich, 2010; Kelley & Kelley, 2019) and are considered the strongest form of data with the fewest biases (Haidich, 2010). In humans, the impacts of diet and lifestyle on the gut microbiota have been assessed using a meta‐analysis approach (Mancabelli et al., 2017). However, there has been no such attempt to do this in the dog. Therefore, this meta‐analysis aimed to evaluate the impacts of dietary protein and fat content on the composition of fecal microbiota in the dog. The hypothesis for this meta‐analysis was that the fecal microbiota would be significantly altered based on the level of protein or fat in the diets. Furthermore, it was hypothesized that performing a meta‐analysis of the data would generate novel insights into the relationship between diet and fecal microbiota.

## 2 MATERIALS AND METHODS

### 2.1 Study protocol

This study has been reported in accordance with the Preferred Reporting Items for Systematic Reviews and Meta‐Analyses (PRISMA) statement (Moher et al., 2009). The project was conceptualized in March 2020, and the protocol was agreed upon in advance before proceeding, with a final review of the literature undertaken in July 2022.

### 2.2 Diet classifications

Diet groupings (low, moderate, high, or supra) were assigned based on the Association of American Feed Control Officials (AAFCO, 2019) recommended minimum crude protein (18% DM) and crude fat (5% DM) content for healthy adult dogs' maintenance. For ease of comparison with AAFCO (2019), Fédération Européenne de l'Industrie des Aliments pour Animaux Familiers (FEDIAF) (2018), and the National Research Council (NRC) (2006), all protein and fat levels were standardized as % DM. Interquartile (IQ) ranges were established for each macronutrient and used to determine the cut‐off between the groups. For example, the first IQ range was used to determine the cut‐off for the low group, rounded to the nearest 5% (Table 1). The minimum dietary protein content as recommended by FEDIAF, NRC, and AAFCO was used as the lowest value for the low group (18% DM) (AAFCO, 2019; FEDIAF, 2018; NRC, 2006).

**Table 1:** Crude protein “protein” and crude fat “fat” diet classifications used to determine the impact of dietary macronutrients on the fecal microbiome of the dog.

Diet classification	Protein	Fat
Low	18% ≤ *x* ≤ 25% DM	5% ≤ *x* ≤ 15% DM
Moderate	25% < *x* ≤ 30% DM	15% < *x* ≤ 20% DM
High	30% < *x* ≤ 45% DM	20% < *x* ≤ 30% DM
Supra	*x* > 45% DM	*x* > 30% DM

Abbreviation: DM, dry matter.

### 2.3 Information sources and searches

A search of the scientific literature for publications that analyzed the fecal microbiota of dogs and provided dietary information was conducted. Online resources searched included OVID databases (Medline, BIOSIS, Food Science and Technology Abstracts [FSTA], CAB Abstracts), Scopus, and PubMed. The search terms initially used were broad: “dog,” “canine,” “diet,” “feeding trial,” “intervention,” “f(a)ecal,” “gut,” “microbiome,” “microbiota,” “microflora,” “microbial,” and “bacterial community” were used to assess the number of studies that could be included and if a meta‐analysis was a viable endeavor. The search terms “gut” and “f(a)ecal” were found to produce similar results, wherein fecal also produced more in vitro‐based publications, although both were dropped for the use of “diet,” which gathered more publications with a focus on dietary changes, and thus, the dietary profiles were more frequently included in the publication. The initial search was conducted from April to June 2020, and a full search of all the databases with these keywords was performed in July 2020. Subsequently, the databases were reassessed quarterly between July 2020 and July 2022 for new publications.

### 2.4 Eligibility criteria

**Figure 1:**
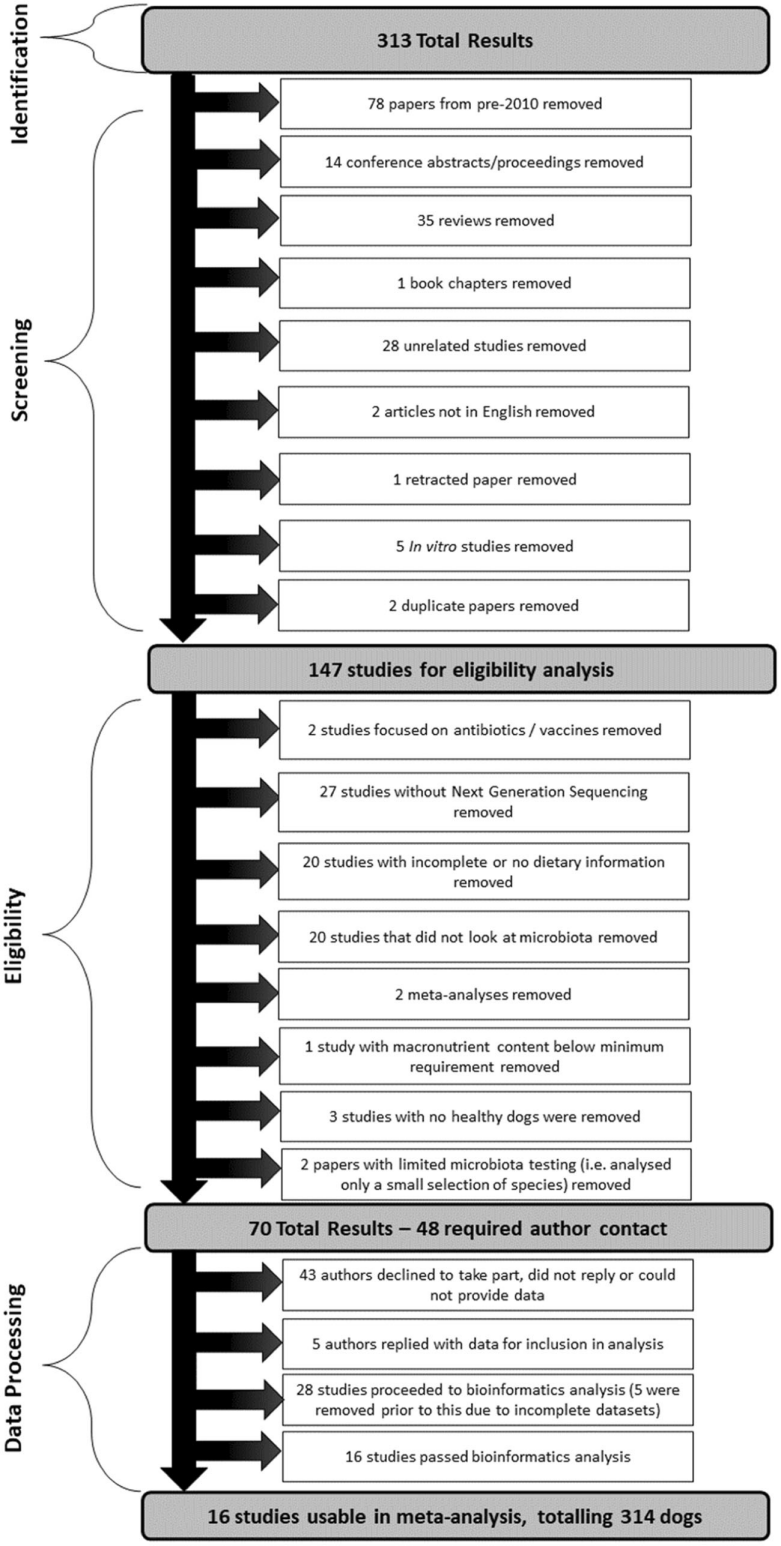
Meta‐analysis publication inclusion/exclusion workflow.

A “publication” was defined as a stand‐alone piece of published work. Publications were analyzed for eligibility as per criteria summarized in Figure 1.

A list of all publications assessed for this meta‐analysis and reasons (if any) for being excluded are shown in Supplementary Data 1 for DOI: 10.1002/mbo3.1404 (figshare.com). In brief, any data arising from dogs with intestinal disorders (e.g., inflammatory bowel disease, chronic enteropathy, food‐responsive diarrhea) and obesity (including weight loss studies) were excluded from this study. However, data from dogs suffering from any nongut‐related illness (e.g., skin problems) were included. Studies that had protein or fat below the required minimum levels established by AAFCO were excluded. Studies, where dietary macronutrient profiles were incomplete but missing values could be calculated were included.

Food intake was not analyzed or included as a factor in assessing the impacts of dietary nutrients on fecal microbial composition. Husbandry status (e.g., kennel, pet, working) and social housing (e.g., individual, paired, pets with and without other pets) were noted.

Studies were included if they analyzed fecal microbial composition using next‐generation sequencing (i.e., 454 pyrosequencing, Illumina 16S ribosomal RNA [rRNA] gene amplicon sequencing, and/or Illumina whole‐genome sequencing), and there was no exclusion based on the variable region of the 16S rRNA gene analyzed, though this is included in Table A1. The methods of extraction of nucleic acids and 16S rRNA gene analysis were all included with metadata tabulation. Studies published from 2010 onwards were included in the analyses.

#### 2.4.1 Study selection and data collection

All publications identified from the electronic searches were reviewed and assessed for eligibility in a standardized, unblinded manner (Figure 1). This was performed quarterly between July 2020 and July 2022. Access rights from both Massey University and AgResearch were used to access the material, and all eligible publications were able to be acquired through this process. All variables listed within each publication were tabulated into a spreadsheet (Microsoft Excel version Office 365; Microsoft).

A total of 313 publications were initially screened. Seventy‐eight papers from pre‐2010 were removed, and additional copies of two duplicated papers were removed. The remaining 231 papers were assessed and are detailed further in Supplementary Data 1 for DOI: 10.1002/mbo3.1404 (figshare.com). Of these, 147 papers met initial inclusion criteria and were then evaluated for eligibility, of which 70 of these papers were deemed eligible (Figure 1). Forty‐eight authors were contacted to request data access using a template email that was agreed upon in advance by the research team and that highlighted any potential conflicts of interest. If there was no response after 2 weeks, contact authors and/or primary authors were contacted again. If no response was obtained from either author or the request was refused, the study was deemed unavailable. A total of 23 publications had complete diet information and had data deposited in publicly accessible archives (e.g., European Nucleotide Archive, Mendeley, National Centre for Biotechnology Information) and met all other inclusion criteria. An additional five publications were included from authors who responded to data queries, which provided a total of 28 publications for analysis, consisting of 74 dietary treatments. Each publication was assigned a unique ID number (Table 2).

**Table 2:** Meta‐data of publications used to assess the effects of dietary crude protein (protein) and/or crude fat (fat) on the composition of the fecal microbiome in the domestic dog.

Study ID	Authors	Reference	Overall number of dogs	Diet ID	Reported protein content (% DM)	Protein treatment	Reported fat (% DM)	Fat treatment	Number of dogs on a diet
ID9	Bermingham et al.^a^	Bermingham et al. (unpublished)	15	Diet 1	39.1	High^b^	19.8	Moderate^c^	8
				Diet 2	24.7	Moderate	12.3	Low	7
ID18	Scarsella et al.	Scarsella et al. (2020)	8	Diet 1	29.1	Moderate	17.3	Moderate	8
ID19	Sandri et al.	Sandri et al. (2020)	27	Diet 1	29.7	Moderate	21.7	High	9
				Diet 2	28.9	Moderate	21.7	High	9
				Diet 3	30.1	High	22.8	High	9
ID23	Kirchoff et al.	Kirchoff et al. (2019)	31	Diet 1	27.8	Moderate	15.6	Moderate	30
				Diet 2	32.2	High	19.4	Moderate	1
ID24	Pilla et al.	Pilla et al. (2019)	8	Diet 1	22.7	Low	10.8	Low	8
ID25	Sandri et al.	Sandri et al. (2019)	8	Diet 1	27.2	Moderate	19.2	Moderate	8
				Diet 2	26.0	Moderate	19.0	Moderate	8
				Diet 3	23.9	Low	15.2	Moderate	8
ID27	Jarett et al.	Jarett et al. (2019)	32	Diet 1	26.2	Moderate	14.2	Low	8
				Diet 2	27.4	Moderate	16.0	Moderate	8
				Diet 3	26.7	Moderate	14.7	Low	8
				Diet 4	25.8	Moderate	14.1	Low	8
ID28	Bresciani et al.	Bresciani et al. (2018)	14	Diet 1	22.0	Low	13.6	Low	14
ID29	Coelho et al.	Coelho et al. (2018)	32	Diet 1	27.3	Moderate	17.5	Moderate	16
				Diet 2	53.9	Supra	18.4	Moderate	16
				Diet 3	30.0	Moderate	18.2	Moderate	32
ID30	Schauf et al.	Schauf et al. (2018)	12	Diet 1	28.5	Moderate	10.9	Low	12
				Diet 2	32.5	High	23.2	High	12
ID32	Qinghong et al.	Li et al. (2017)	32	Diet 1	53.3	Supra	15.1	Moderate	15
				Diet 2	27.6	Moderate	15.7	Moderate	16
				Diet 3	30.5	High	17.2	High	16
ID38	Kilburn et al.	Kilburn et al. (2020)	8	Diet 1	46.9	Supra	32.1	Supra	8
				Diet 2	42.7	High	37.2	Supra	8
				Diet 3	40.0	High	41.9	Supra	8
				Diet 4	38.2	High	46.5	Supra	8
ID39	Beloshapka et al.	Beloshapka et al. (2021)	7	Diet 1	37.7	High	13.2	Low	7
ID43	Moinard et al.	Moinard et al. (2020)	24	Diet 1	30.6	High	34.5	Supra	24
ID44	Martínez‐López et al.	Martínez‐López et al. (2021)	46	Diet 1	21.1	Low	6.6	Low	46
				Diet 2	19.0	Low	14.4	Low	46
				Diet 3	19.2	Low	8.7	Low	46
ID45	Eisenhauer et al.	Eisenhauer et al. (2019)	10	Diet 1	21.3	Low	10.3	Low	10
				Diet 2	40.9	High	10.9	Low	10
				Diet 3	21.6	Moderate	10.1	Low	10
				Diet 4	39.9	High	10.5	Low	10
				Diet 5	21.9	Moderate	9.9	Low	10

*Note*: Diet ID indicates the number of diets used within each study; treatment refers to the classification of the % dry matter (DM) protein or fat content in the diet (low, moderate, high, or supra).

^a^ Unpublished study.

^b^ Low protein was classified as <25% crude protein content by dry matter (% DM) analysis. Moderate protein was between 25% and 30% DM. High protein was between 30% and 45% DM. Any reported crude protein content higher than 45% DM was classed as supra protein.

^c^ Low fat was classified as <15% DM dietary fat content. Moderate fat was between 15% and 20% DM. High fat was between 20% and 30% DM. Any reported dietary fat content higher than 30% DM was classed as supra fat.

### 2.5 Bioinformatics analysis

Sequence reads from each publication were downloaded from the relevant data deposition, and the sequencing reads from individual studies were processed within each study using one of the following pipelines. The raw reads produced by the sequencing instrument were merged using the program FLASH2 v2.2.00 (Magoč & Salzberg, 2011). Merged reads were then quality trimmed using Trimmomatic v0.38 (Bolger et al., 2014). The trimmed reads were reformatted as fasta, and the read headers were modified to include the sample name. All reads were compiled into a single file, and Mothur v1.45.2 (Schloss et al., 2009) was used to remove reads with homopolymers longer than 10 nucleotides (nt) and to collapse the reads into unique representatives. The collapsed reads were clustered using Swarm v2 (Mahé et al., 2014). The clustered reads were filtered based on their abundance, keeping representatives that were (i) present in one sample with a relative abundance of >0.1%, (ii) present in >2% of the samples with a relative abundance of >0.01%, or (iii) present in 5% of the samples at any abundance level. The selected representatives were annotated using QIIME 2 v2017.4 (Caporaso et al., 2010) with the SILVA database v138 (Quast et al., 2013). The annotated tables were then used for downstream statistical analysis.

Whole‐genome shotgun sequencing (WGS) sequencing reads derived from ribosomal DNA was extracted using Metaxa version 2.1.3 (Bengtsson‐Palme et al., 2015) and aligned to the SILVA 138 ribosomal RNA gene database (Quast et al., 2013) using the “assign_taxonomy.py” script from qiime version 1.9 (Caporaso et al., 2010).

Twelve papers were deemed to have insufficient data quality for further analysis. Thus, 16 studies were included in the final meta‐analysis, consisting of samples from 314 dogs.

### 2.6 Statistical analyses

**Figure 2:**
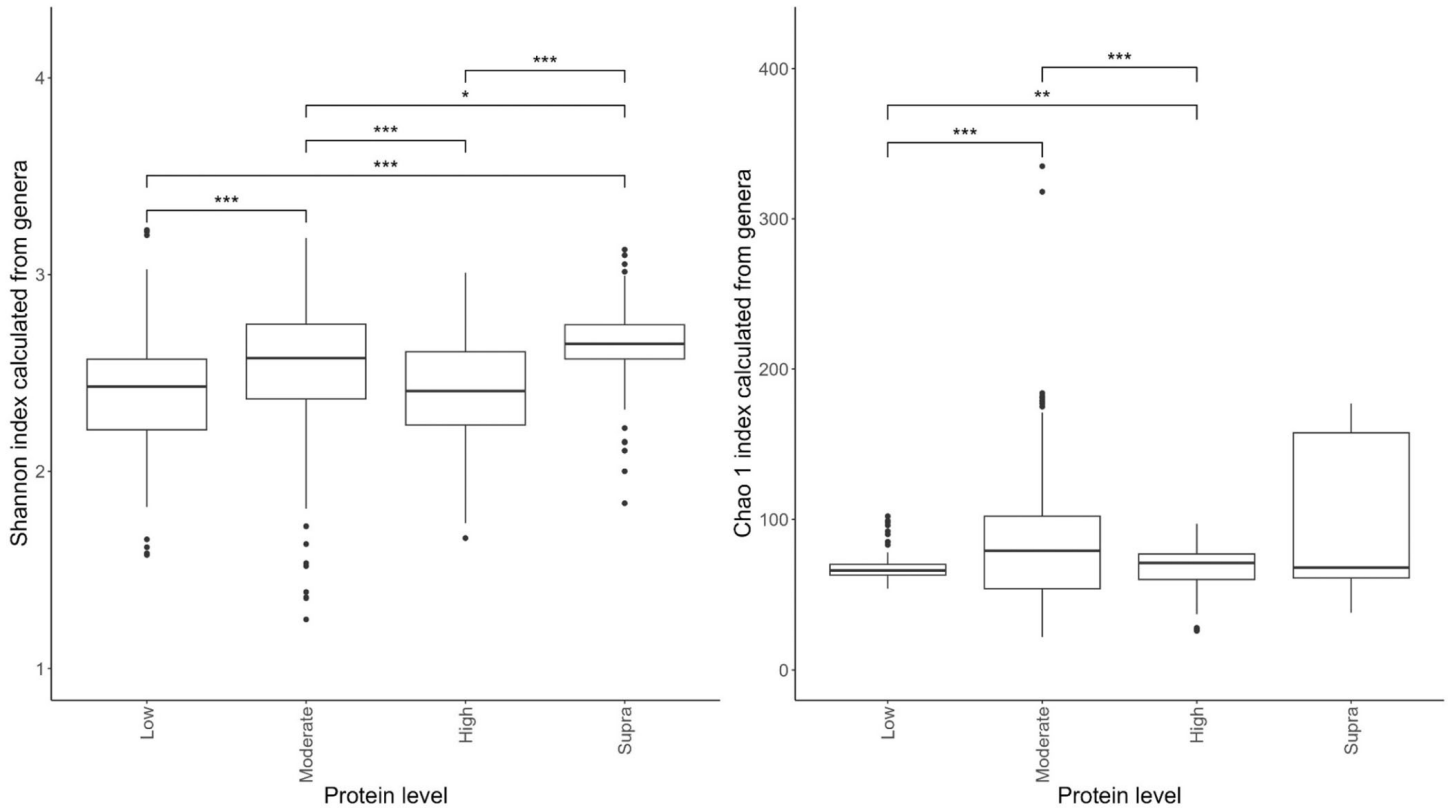
Alpha diversity using the Shannon index (Figure 2a) and Chao 1 index (Figure 2b) of the fecal microbiome of the dog fed differing levels of dietary protein. A total of seven diets were classed as low‐protein (≤25% DM; *n* = 178 dogs), 17 were moderate‐protein (25%–30% DM; *n* = 208 dogs), 12 were high‐protein (30%–45% DM; *n* = 120 dogs), and three were supra‐protein diets (≥45% DM; *n* = 39 dogs). Statistical differences between protein classifications are denoted by an asterisk (*), where *p < 0.05; **p < 0.01; ***p < 0.001. Circles denote outliers. Boxes represent the interquartile range between the first and third quartiles. The thick black line inside the box denotes the median.

The data were loaded into R (version 4.1.1; RStudio). The studies were evaluated for suitability by viewing the rarefaction curve produced by the “vegan” R package version 2.5‐7 (Dixon, 2003). Proportions of genera were first converted into pseudocounts based on the lowest proportion greater than 0 in the study that the sample belonged to and dividing 1 by it, giving a factor to multiply all the proportions by to resurrect relative counts. All studies were found to have sufficient counts. The diversity indices were also calculated from the proportions, and nonparametric two‐sample Kolmogorov–Smirnov tests were performed on the diversity indices to assess the significance between protein and fat classifications: the results of which are displayed in Figure 2.

**Figure A1:**
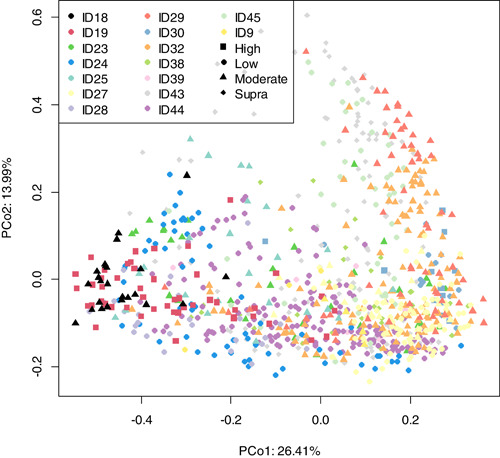
Principal coordinate analysis plot of bacteria, colored by study, with points shaped by fat level.

**Figure A2:**
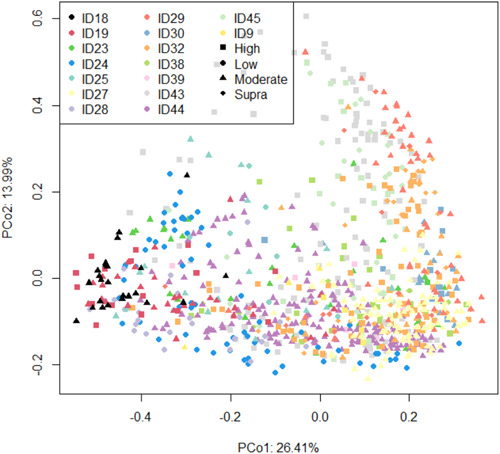
Principal coordinate analysis plot of bacteria, colored by study, with points shaped by protein level.

Principal coordinate analysis (PCoA) was performed using the “pcoa” function in the “ape” R package version 5.5 (Paradis & Schliep, 2018) on the Bray–Curtis dissimilarity matrices derived using the “vegdist” function of the “vegan” R package version 2.5‐7 (Dixon, 2003) from the proportions of the genera from each of the studies. The “anosim” function of the “vegan” R package was used to perform an analysis of similarities (ANOSIM) on the Bray–Curtis dissimilarity matrices with default settings and the protein and fat levels as the groupings. The points were colored by the “level” of protein in the diet. An alternative set of PCoA plots was produced to assess the effect of the study on the ordination (Figures A1 and A2). Partial least squares‐discriminant analysis (PLS‐DA) was performed on the proportions of genera using either the fat level or protein level as a response, using the “opls” function in the “rop ls” R package, version 1.24.0 (Thévenot et al., 2015). Random forest analysis was also performed on the proportions of genera using either the fat level or protein level as a response using the “randomForest” function in the “RandomForest” R package, version 4.6‐14 (Liaw & Wiener, 2007). Finally, permutation analysis of variance (ANOVA) was performed on the proportions of genera using either the fat or protein level as a response with the “aovp” function from the “lmPerm” R package, version 2.1.0 (Metsalu & Vilo, 2015). Genstat (19th Edition; VSN International) was used to perform one‐way ANOVAs on the diet metadata to assess for differences between defined diet groupings, with Tukey's posthoc analysis to determine differences between groups.

## 3 RESULTS

### 3.1 Metadata

After the literature search, author contact, and bioinformatics analysis, a total of 16 studies were eligible for inclusion in the meta‐study. This produced a data set that comprised 314 dogs (Table 2). Due to different study designs incorporating different feeding regimens (i.e., crossover studies or individual comparisons), there were 545 data sets associated with diet, which are also detailed in Table 2.

### 3.2 Associations between protein level and the fecal microbiome

Of the diets included in the meta‐analysis, seven were classed as low‐protein (≤25% DM; *n* = 178 dogs), 17 were moderate‐protein (25%–30% DM; *n* = 208 dogs), 12 were high‐protein (30%–45% DM; *n* = 120 dogs), and three were supra‐protein diets (≥45% DM; *n* = 39 dogs).

The effects of protein classification on fecal diversity measures are shown in Figure 2. Despite some overlaps between dietary treatments, protein content affected (*p* < 0.001) alpha diversity (Shannon index) of the fecal microbiome. Alpha‐diversity indices were highest in the moderate‐protein classification and lowest in the supra‐protein classification (Figure 2a). The total genus richness (Chao 1 index) of the fecal microbiome was affected by dietary crude protein content in the dog (*p* < 0.001; Figure 2b). The bacterial richness was highest in the supra‐protein and lowest in the low‐protein classification.

**Figure 3:**
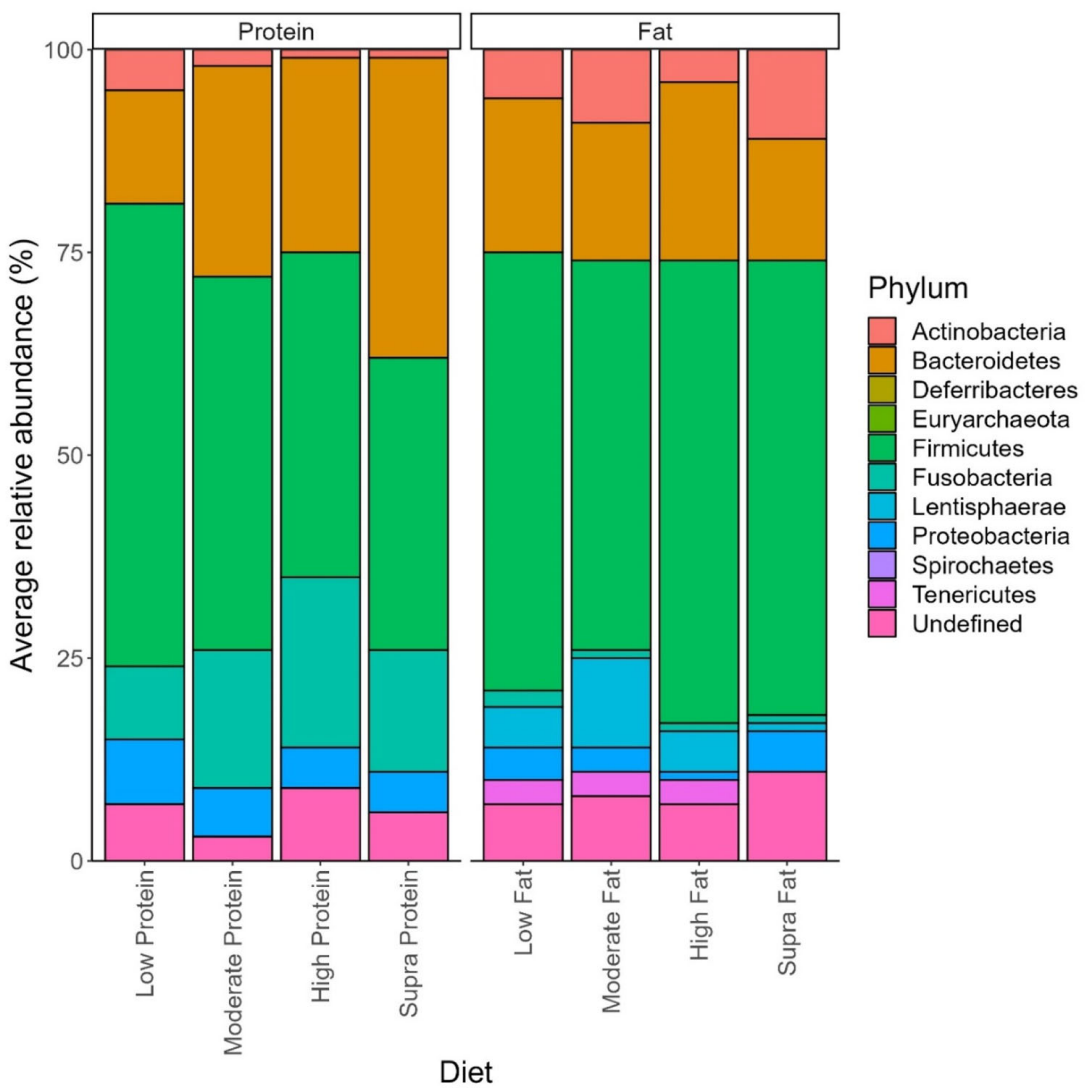
Effect of dietary protein and dietary fat on the relative abundance of phyla observed in the feces of dogs.

The effect of protein on the abundance of phyla is shown in Figure 3. Overall, 226 genera were identified in this data set, of which 215 showed small but significant (*p* < 0.05) differences depending on dietary protein levels (Table 3). *Peptoclostridium* (11.44 ± 0.82% sequence reads), *Bacteroides* (10.46 ± 0.88% sequence reads), *Fusobacterium* (9.30 ± 0.74% sequence reads), *Blautia* (8.45 ± 0.64% sequence reads), and *Streptococcus* (6.14 ± 1.02% of sequence reads) were the dominant genera observed in dogs assigned to the low‐protein diet (Table 3). In the dogs assigned to the moderate‐protein diet, *Fusobacterium* (16.34 ± 0.51% of sequence reads), *Bacteroides* (10.52 ± 0.41% of sequence reads), *Peptoclostridium* (8.41 ± 0.35% of sequence reads), *Prevotella* (7.83 ± 0.53% of sequence reads), and *Blautia* (5.51 ± 0.254% of sequence reads) were the most abundant bacterial genera observed (Table 3). For the dogs assigned to the high‐protein classification, the dominant bacterial genera in terms of relative abundance were *Fusobacterium* (20.01 ± 0.737% of sequence reads), *Bacteroides* (11.04 ± 0.50% of sequence reads), *Peptoclostridium* (9.88 ± 0.573% of sequence reads), uncultured bacterium (7.78 ± 0.48% of sequence reads), and *Allobaculum* (5.16 ± 0.45% of sequence reads) (Table 3). Finally, *Fusobacterium* (14.94 ± 0.85% of sequence reads), *Bacteroides* (13.91 ± 0.94% of sequence reads), *Prevotella* (10.55% ± 1.14% of sequence reads), *Peptoclostridium* (8.93 ± 1.20% of sequence reads), and *Alloprevotella* (7.33 ± 0.85% of sequence reads) were the dominant genera observed in the dogs assigned to the supra‐protein classification (Table 3).

**Table 3:** Relative abundances (% of sequence reads) averaging above 0.001% of sequence reads in one treatment of the fecal microbiome of the dog fed differing levels of dietary protein.

Phylum	Genus	Low protein, mean ± SEM	Moderate protein, mean ± SEM	High protein, mean ± SEM	Supra protein, mean ± SEM	FDR‐adjusted, *p* value
Actinobacteria	*Adlercreutzia*	0.0 ± 0.0a	0.0 ± 0.0b	0.0 ± 0.0a	0.0 ± 0.0b	<0.001
	*Atopobium*	‐	0.0 ± 0.0	‐	0.0 ± 0.0	<0.001
	*Bifidobacterium*	1.3 ± 0.3a	0.7 ± 0.1b	0.5 ± 0.1b	0.3 ± 0.1b	<0.001
	*Collinsella*	3.0 ± 0.3a	1.3 ± 0.1b	0.7 ± 0.1b	0.4 ± 0.1b	<0.001
	*Coriobacteriaceae UCG.002*	0.1 ± 0.0a	0.0 ± 0.0b	0.1 ± 0.0a	0.0 ± 0.0ab	<0.001
	*Corynebacterium*	0.0 ± 0.0	0.0 ± 0.0	0.0 ± 0.0	0.0 ± 0.0	<0.001
	*Denitrobacterium*	0.0 ± 0.0a	0.0 ± 0.0b	0.0 ± 0.0b	0.0 ± 0.0b	<0.001
	*Olsenella*	0.1 ± 0.0a	0.0 ± 0.0b	0.1 ± 0.0ab	0.0 ± 0.0ab	<0.001
	*Slackia*	0.1 ± 0.0b	0.2 ± 0.0a	0.1 ± 0.0b	0.1 ± 0.0b	<0.001
	*Trueperella*	‐	0.0 ± 0.0	0.1 ± 0.1	0.0 ± 0.0	<0.001
Bacteroidetes	*Alistipes*	0.0 ± 0.0a	0.0 ± 0.0b	0.0 ± 0.0a	0.0 ± 0.0a	<0.001
	*Alloprevotella*	1.5 ± 0.2c	4.0 ± 0.2b	4.2 ± 0.3b	7.3 ± 0.9a	<0.001
	*Bacteroides*	10.5 ± 0.9b	10.5 ± 0.4b	11.0 ± 0.5b	13.9 ± 0.9a	<0.001
	*Barnesiella*	‐	0.0 ± 0.0b	‐	0.0 ± 0.0a	<0.001
	*Muribaculaceae_ge*	0.4 ± 0.1b	0.6 ± 0.1b	2.3 ± 0.3a	2.2 ± 0.5a	<0.001
	*Myroides*	‐	‐	0.0 ± 0.0	‐	0.191
	*Odoribacter*	0.0 ± 0.0b	0.0 ± 0.0a	0.0 ± 0.0b	0.0 ± 0.0a	<0.001
	*Parabacteroides*	0.1 ± 0.0c	0.1 ± 0.0b	0.2 ± 0.0a	0.2 ± 0.0a	<0.001
	*Paraprevotella*	0.0 ± 0.0	0.0 ± 0.0	0.0 ± 0.0	0.0 ± 0.0	0.235
	*Porphyromonas*	‐	0.0 ± 0.0	0.0 ± 0.0	0.0 ± 0.0	<0.001
	*Prevotella*	1.3 ± 0.2c	7.8 ± 0.5a	4.8 ± 0.5b	10.6 ± 1.1a	<0.001
	*Prevotellaceae Ga6A1 group*	0.6 ± 0.1c	2.4 ± 0.2a	1.7 ± 0.2b	2.3 ± 0.6ab	<0.001
	*Prevotellaceae NK3B31 group*	‐	0.0 ± 0.0b	0.0 ± 0.0b	0.1 ± 0.1a	<0.001
	*Prevotellaceae UCG.001*	0.0 ± 0.0bc	0.0 ± 0.0b	0.0 ± 0.0c	0.0 ± 0.0a	<0.001
	*Prevotellaceae UCG.003*	0.0 ± 0.0	0.0 ± 0.0	0.0 ± 0.0	0.1 ± 0.1	<0.001
	*Rikenellaceae RC9 gut group*	0.1 ± 0.0b	0.1 ± 0.0b	0.1 ± 0.0b	0.2 ± 0.0a	<0.001
Deferribacteres	*Mucispirillum*	0.0 ± 0.0b	0.0 ± 0.0a	0.0 ± 0.0b	0.0 ± 0.0ab	<0.001
Euryarchaeota	*Methanobrevibacter*	‐	0.0 ± 0.0	0.0 ± 0.0	0.0 ± 0.0	<0.001
	*Methanosphaera*	0.0 ± 0.0b	0.0 ± 0.0b	0.1 ± 0.0a	0.0 ± 0.0b	<0.001
Firmicutes	*Acidaminococcus*	‐	0.0 ± 0.0	0.0 ± 0.0	0.0 ± 0.0	<0.001
	*Agathobacter*	‐	0.0 ± 0.0b	‐	0.0 ± 0.0a	<0.001
	*Allisonella*	0.0 ± 0.0a	0.0 ± 0.0ab	0.0 ± 0.0b	0.0 ± 0.0a	<0.001
	*Allobaculum*	2.4 ± 0.5b	1.6 ± 0.2b	5.2 ± 0.5a	2.6 ± 0.3b	<0.001
	*Anaerofilum*	0.1 ± 0.0a	0.0 ± 0.0c	0.0 ± 0.0b	0.0 ± 0.0bc	<0.001
	*Anaerofustis*	‐	0.0 ± 0.0b	0.0 ± 0.0b	0.2 ± 0.1a	<0.001
	*Anaerostipes*	0.0 ± 0.0	0.0 ± 0.0	‐	0.0 ± 0.0	<0.001
	*Anaerovibrio*	‐	0.0 ± 0.0	0.0 ± 0.0	0.0 ± 0.0	<0.001
	*Anaerovoracaceae ge*	0.4 ± 0.1	0.3 ± 0.0	0.4 ± 0.0	0.4 ± 0.1	<0.001
	*Bacillus*	0.1 ± 0.0a	0.0 ± 0.0b	‐	0.0 ± 0.0b	<0.001
	*Blautia*	8.5 ± 0.6a	5.5 ± 0.3b	4.5 ± 0.3c	3.1 ± 0.3c	<0.001
	*Butyricicoccus*	0.1 ± 0.0bc	0.2 ± 0.0a	0.1 ± 0.0c	0.1 ± 0.0ab	<0.001
	*Candidatus Arthromitus*	0.0 ± 0.0ab	0.0 ± 0.0a	0.0 ± 0.0b	0.0 ± 0.0b	<0.001
	*Candidatus Soleaferrea*	‐	0.0 ± 0.0b	0.0 ± 0.0b	0.0 ± 0.0a	<0.001
	*Candidatus Stoquefichus*	0.0 ± 0.0b	0.0 ± 0.0b	0.1 ± 0.0a	0.0 ± 0.0b	<0.001
	*Caproiciproducens*	‐	0.0 ± 0.0b	‐	0.0 ± 0.0a	<0.001
	*Carnobacterium*	0.0 ± 0.0b	0.0 ± 0.0a	0.0 ± 0.0b	0.0 ± 0.0b	<0.001
	*Catellicoccus*	‐	0.0 ± 0.0	‐	‐	<0.001
	*Catenibacterium*	0.9 ± 0.2bc	1.0 ± 0.1b	0.5 ± 0.1c	1.7 ± 1.0a	<0.001
	*Catenisphaera*	0.0 ± 0.0b	0.1 ± 0.0b	0.1 ± 0.0a	0.1 ± 0.0b	<0.001
	*Cellulosilyticum*	0.0 ± 0.0a	0.0 ± 0.0a	0.1 ± 0.0a	0.1 ± 0.0a	0.002
	*CHKCI001*	0.0 ± 0.0a	0.0 ± 0.0b	0.0 ± 0.0b	0.0 ± 0.0b	<0.001
	*Christensenellaceae_ge*	0.0 ± 0.0	0.0 ± 0.0	0.0 ± 0.0	0.0 ± 0.0	0.224
	*Christensenellaceae R.7 group*	0.0 ± 0.0b	0.0 ± 0.0a	0.0 ± 0.0b	0.0 ± 0.0a	<0.001
	*Clostridioides*	0.0 ± 0.0b	0.0 ± 0.0b	0.0 ± 0.0b	0.1 ± 0.0a	<0.001
	*Clostridium sensu stricto 1*	2.5 ± 0.4	2.2 ± 0.2	1.7 ± 0.2	1.0 ± 0.2	0.087
	*Clostridium sensu stricto 13*	0.0 ± 0.0b	0.0 ± 0.0a	0.0 ± 0.0b	0.0 ± 0.0b	<0.001
	*Clostridium sensu stricto 18*	‐	0.0 ± 0.0b	‐	0.0 ± 0.0a	<0.001
	*Clostridium sensu stricto 2*	‐	0.0 ± 0.0	‐	0.0 ± 0.0	<0.001
	*Clostridium sensu stricto 7*	‐	0.2 ± 0.1	‐	0.0 ± 0.0	<0.001
	*Colidextribacter*	0.0 ± 0.0b	0.0 ± 0.0a	0.0 ± 0.0b	0.0 ± 0.0a	<0.001
	*Coprobacillus*	‐	0.0 ± 0.0a	0.0 ± 0.0b	0.0 ± 0.0ab	<0.001
	*Coprococcus*	0.0 ± 0.0c	0.0 ± 0.0b	0.0 ± 0.0bc	0.0 ± 0.0a	<0.001
	*Dialister*	0.0 ± 0.0a	0.0 ± 0.0b	0.0 ± 0.0ab	0.0 ± 0.0ab	<0.001
	*Dorea*	0.0 ± 0.0c	0.0 ± 0.0b	0.0 ± 0.0c	0.1 ± 0.0a	<0.001
	*Dubosiella*	0.2 ± 0.1b	0.2 ± 0.0b	0.5 ± 0.0a	0.3 ± 0.1ab	<0.001
	*Enterococcus*	2.0 ± 0.5a	0.3 ± 0.1b	0.0 ± 0.0b	0.0 ± 0.0b	<0.001
	*Epulopiscium*	0.1 ± 0.0	0.1 ± 0.0	0.1 ± 0.0	0.0 ± 0.0	0.307
	*Erysipelatoclostridium*	0.2 ± 0.0	0.2 ± 0.0	0.1 ± 0.0	0.1 ± 0.0	0.308
	*Erysipelotrichaceae_ge*	0.0 ± 0.0ab	0.0 ± 0.0a	0.0 ± 0.0b	0.0 ± 0.0ab	<0.001
	*Erysipelotrichaceae UCG.003*	0.5 ± 0.1a	0.2 ± 0.0b	0.1 ± 0.0b	0.0 ± 0.0b	<0.001
	*Eubacterium*	0.0 ± 0.0	0.0 ± 0.0	0.0 ± 0.0	0.0 ± 0.0	<0.001
	*Faecalibacterium*	1.9 ± 0.3	2.2 ± 0.1	2.4 ± 0.2	2.2 ± 0.3	0.002
	*Faecalibaculum*	0.1 ± 0.0b	0.1 ± 0.0b	0.1 ± 0.0a	0.1 ± 0.0ab	<0.001
	*Faecalicoccus*	‐	0.0 ± 0.0a	‐	0.0 ± 0.0a	<0.001
	*Faecalitalea*	0.1 ± 0.0a	0.0 ± 0.0b	0.0 ± 0.0b	0.0 ± 0.0b	<0.001
	*Family XIII AD3011 group*	0.0 ± 0.0	0.0 ± 0.0	0.0 ± 0.0	0.0 ± 0.0	<0.001
	*Family XIII UCG.001*	0.0 ± 0.0bc	0.0 ± 0.0b	‐	0.0 ± 0.0a	<0.001
	*Flavonifractor*	0.0 ± 0.0a	0.0 ± 0.0b	‐	0.0 ± 0.0b	<0.001
	*Fournierella*	0.0 ± 0.0c	0.1 ± 0.0b	0.1 ± 0.0b	0.2 ± 0.0a	<0.001
	*Fusibacter*	‐	0.0 ± 0.0	0.0 ± 0.0	0.0 ± 0.0	<0.001
	*Fusicatenibacter*	0.0 ± 0.0a	0.0 ± 0.0b	0.0 ± 0.0b	0.0 ± 0.0b	<0.001
	*GCA.900066575*	0.0 ± 0.0b	0.1 ± 0.0a	0.0 ± 0.0b	0.0 ± 0.0ab	<0.001
	*Hathewaya*	‐	0.0 ± 0.0	‐	0.0 ± 0.0	<0.001
	*Holdemanella*	0.9 ± 0.1	0.7 ± 0.1	0.5 ± 0.1	0.4 ± 0.1	0.806
	*Howardella*	0.0 ± 0.0b	0.0 ± 0.0a	0.0 ± 0.0b	0.0 ± 0.0ab	<0.001
	*Hungatella*	0.0 ± 0.0	0.0 ± 0.0	0.0 ± 0.0	0.0 ± 0.0	0.047
	*Ileibacterium*	0.0 ± 0.0b	0.2 ± 0.0b	0.3 ± 0.1a	0.3 ± 0.1ab	<0.001
	*Intestinibacter*	0.0 ± 0.0c	0.1 ± 0.0b	0.0 ± 0.0c	0.2 ± 0.1a	<0.001
	*Intestinimonas*	0.0 ± 0.0b	0.0 ± 0.0a	0.0 ± 0.0a	0.0 ± 0.0a	<0.001
	*Lachnoclostridium*	1.5 ± 0.3a	0.5 ± 0.0b	0.5 ± 0.0b	0.6 ± 0.1b	<0.001
	*Lachnospira*	0.3 ± 0.1a	0.1 ± 0.0c	0.2 ± 0.0b	0.0 ± 0.0c	<0.001
	*Lachnospiraceae AC2044 group*	‐	0.0 ± 0.0a	0.0 ± 0.0b	0.0 ± 0.0ab	<0.001
	*Lachnospiraceae FCS020 group*	0.0 ± 0.0	0.0 ± 0.0	0.0 ± 0.0	0.0 ± 0.0	<0.001
	*Lachnospiraceae ge*	5.5 ± 0.5a	2.6 ± 0.1b	2.5 ± 0.1b	1.9 ± 0.3b	<0.001
	*Lachnospiraceae ND3007 group*	‐	0.0 ± 0.0b	0.0 ± 0.0b	0.0 ± 0.0a	<0.001
	*Lachnospiraceae NK3A20 group*	‐	0.0 ± 0.0b	‐	0.0 ± 0.0a	<0.001
	*Lachnospiraceae NK4A136 group*	0.2 ± 0.0bc	0.2 ± 0.0b	0.2 ± 0.0c	0.4 ± 0.1a	<0.001
	*Lachnospiraceae UCG.003*	0.0 ± 0.0b	0.0 ± 0.0a	0.0 ± 0.0b	0.0 ± 0.0b	0.006
	*Lachnospiraceae UCG.004*	0.0 ± 0.0c	0.0 ± 0.0b	0.0 ± 0.0c	0.0 ± 0.0a	<0.001
	*Lachnospiraceae XPB1014 group*	‐	0.0 ± 0.0b	0.0 ± 0.0bc	0.0 ± 0.0a	<0.001
	*Lactobacillus*	1.6 ± 0.5b	3.5 ± 0.4a	4.3 ± 0.4a	2.7 ± 0.7ab	<0.001
	*Lactococcus*	0.0 ± 0.0ab	0.0 ± 0.0a	0.0 ± 0.0b	0.0 ± 0.0ab	<0.001
	*Leuconostoc*	‐	0.0 ± 0.0a	0.0 ± 0.0b	0.0 ± 0.0b	<0.001
	*Marvinbryantia*	0.6 ± 0.2a	0.1 ± 0.0b	0.1 ± 0.0b	0.0 ± 0.0b	<0.001
	*Megamonas*	3.1 ± 0.5b	4.5 ± 0.3a	0.9 ± 0.1c	2.3 ± 0.3bc	<0.001
	*Megasphaera*	0.1 ± 0.1	0.0 ± 0.0	0.1 ± 0.0	0.2 ± 0.2	<0.001
	*Mitsuokella*	‐	0.0 ± 0.0	0.0 ± 0.0a	0.0 ± 0.0	<0.001
	*Monoglobus*	0.0 ± 0.0b	0.0 ± 0.0a	0.0 ± 0.0b	0.0 ± 0.0ab	<0.001
	*Negativibacillus*	0.2 ± 0.0	0.2 ± 0.0	0.2 ± 0.0	0.2 ± 0.0	<0.001
	*NK4A214 group*	0.0 ± 0.0c	0.0 ± 0.0b	0.0 ± 0.0b	0.0 ± 0.0a	<0.001
	*Oribacterium*	0.0 ± 0.0b	0.0 ± 0.0a	0.0 ± 0.0a	0.1 ± 0.0a	<0.001
	*Oscillibacter*	0.0 ± 0.0c	0.0 ± 0.0b	0.0 ± 0.0c	0.0 ± 0.0a	<0.001
	*Oscillospira*	0.0 ± 0.0c	0.0 ± 0.0a	0.0 ± 0.0bc	0.0 ± 0.0ab	<0.001
	*Paeniclostridium*	‐	0.0 ± 0.0	0.0 ± 0.0	0.0 ± 0.0	<0.001
	*Paludicola*	0.0 ± 0.0bc	0.0 ± 0.0b	0.0 ± 0.0c	0.0 ± 0.0a	<0.001
	*Paraclostridium*	0.1 ± 0.0b	0.3 ± 0.1a	0.2 ± 0.0ab	0.0 ± 0.0b	<0.001
	*Peptoclostridium*	11.4 ± 0.8a	8.4 ± 0.4b	9.9 ± 0.6a	8.9 ± 1.2ab	0.029
	*Peptococcus*	0.2 ± 0.0c	0.8 ± 0.1a	0.5 ± 0.0b	0.3 ± 0.0bc	<0.001
	*Peptoniphilus*	‐	0.6 ± 0.1a	‐	0.0 ± 0.0b	<0.001
	*Peptostreptococcaceae ge*	0.0 ± 0.0ab	0.0 ± 0.0a	0.0 ± 0.0b	0.0 ± 0.0ab	<0.001
	*Peptostreptococcus*	0.1 ± 0.0b	1.1 ± 0.2a	0.2 ± 0.0b	0.3 ± 0.1b	<0.001
	*Phascolarctobacterium*	0.5 ± 0.1c	1.8 ± 0.1a	1.1 ± 0.1b	1.5 ± 0.1ab	<0.001
	*Phocea*	0.0 ± 0.0	0.0 ± 0.0	0.0 ± 0.0	0.0 ± 0.0	<0.001
	*Pygmaiobacter*	0.1 ± 0.0b	0.1 ± 0.0a	0.0 ± 0.0b	0.0 ± 0.0b	<0.001
	*Robinsoniella*	0.1 ± 0.0a	0.0 ± 0.0c	0.0 ± 0.0b	0.0 ± 0.0bc	<0.001
	*Romboutsia*	0.8 ± 0.2	0.6 ± 0.1	0.8 ± 0.1	0.7 ± 0.2	<0.001
	*Roseburia*	0.0 ± 0.0	0.0 ± 0.0	0.0 ± 0.0	0.1 ± 0.0	<0.001
	*Ruminococcus*	0.0 ± 0.0ab	0.0 ± 0.0b	0.0 ± 0.0a	0.0 ± 0.0a	<0.001
	*Sarcina*	0.5 ± 0.1a	0.3 ± 0.1a	0.0 ± 0.0b	0.0 ± 0.0ab	<0.001
	*Sharpea*	‐	0.0 ± 0.0c	0.1 ± 0.0a	0.0 ± 0.0b	<0.001
	*Shuttleworthia*	‐	0.0 ± 0.0b	0.0 ± 0.0c	0.1 ± 0.0a	<0.001
	*Solobacterium*	‐	0.0 ± 0.0ab	0.0 ± 0.0b	0.0 ± 0.0a	<0.001
	*Sporosarcina*	‐	0.0 ± 0.0a	‐	0.0 ± 0.0b	<0.001
	*Staphylococcus*	0.0 ± 0.0a	0.0 ± 0.0b	0.0 ± 0.0ab	0.0 ± 0.0ab	<0.001
	*Streptococcus*	6.1 ± 1.0a	2.2 ± 0.3b	0.6 ± 0.2c	1.6 ± 0.9bc	<0.001
	*Subdoligranulum*	0.1 ± 0.0a	0.0 ± 0.0b	0.0 ± 0.0b	0.0 ± 0.0ab	<0.001
	*Terrisporobacter*	0.9 ± 0.2a	0.1 ± 0.0b	0.2 ± 0.0b	0.2 ± 0.0b	<0.001
	*Turicibacter*	1.2 ± 0.2b	2.0 ± 0.2a	1.0 ± 0.1b	0.6 ± 0.1b	<0.001
	*Tuzzerella*	0.0 ± 0.0c	0.0 ± 0.0b	0.0 ± 0.0b	0.0 ± 0.0a	<0.001
	*Tyzzerella*	0.3 ± 0.1a	0.1 ± 0.0b	0.0 ± 0.0c	0.0 ± 0.0c	<0.001
	*Vagococcus*	‐	0.0 ± 0.0a	‐	0.0 ± 0.0b	<0.001
	*Weissella*	0.0 ± 0.0a	0.0 ± 0.0ab	0.0 ± 0.0b	0.0 ± 0.0b	<0.001
Fusobacteria	*Cetobacterium*	0.2 ± 0.1b	0.3 ± 0.1b	0.6 ± 0.1a	0.4 ± 0.1ab	<0.001
	*Fusobacterium*	9.3 ± 0.7c	16.3 ± 0.5b	20.0 ± 0.7a	14.9 ± 0.9b	<0.001
	*Oceanivirga*	‐	0.0 ± 0.0	0.0 ± 0.0	0.0 ± 0.0	<0.001
	*Streptobacillus*	‐	0.0 ± 0.0	0.1 ± 0.1	0.0 ± 0.0	<0.001
Lentisphaerae	*Victivallis*	0.0 ± 0.0a	0.0 ± 0.0b	0.0 ± 0.0ab	‐	0.062
	*Actinobacillus*	‐	0.0 ± 0.0	0.1 ± 0.1	0.0 ± 0.0	<0.001
	*Aestuariibacter*	0.1 ± 0.0a	0.0 ± 0.0b	0.0 ± 0.0b	0.0 ± 0.0b	<0.001
Proteobacteria	*Anaerobiospirillum*	0.2 ± 0.0c	1.4 ± 0.1a	1.0 ± 0.1b	1.3 ± 0.2ab	<0.001
	*Bosea*	‐	‐	0.0 ± 0.0	0.0 ± 0.0	<0.001
	*Campylobacter*	0.1 ± 0.0a	0.0 ± 0.0b	0.0 ± 0.0b	0.0 ± 0.0b	<0.001
	*Citrobacter*	‐	0.0 ± 0.0	‐	0.0 ± 0.0	<0.001
	*Cupriavidus*	‐	0.0 ± 0.0a	0.0 ± 0.0b	0.0 ± 0.0ab	<0.001
	*Desulfovibrio*	0.0 ± 0.0	0.0 ± 0.0	‐	0.0 ± 0.0	<0.001
	*Enterobacter*	0.5 ± 0.1a	0.0 ± 0.0b	0.0 ± 0.0b	0.0 ± 0.0b	<0.001
	*Escherichia.Shigella*	5.1 ± 0.8a	1.3 ± 0.2b	0.3 ± 0.1c	0.2 ± 0.1c	<0.001
	*Hafnia.Obesumbacterium*	‐	0.0 ± 0.0	‐	‐	<0.001
	*Helicobacter*	0.4 ± 0.1a	0.1 ± 0.0b	0.1 ± 0.0b	0.1 ± 0.0b	<0.001
	*Histophilus*	‐	‐	0.1 ± 0.1a	0.0 ± 0.0a	<0.001
	*Kosakonia*	‐	0.0 ± 0.0	0.0 ± 0.0	0.0 ± 0.0	0.117
	*Parasutterella*	0.9 ± 0.2b	1.1 ± 0.1b	1.9 ± 0.2a	2.1 ± 0.4a	<0.001
	*Plesiomonas*	0.0 ± 0.0	0.1 ± 0.0	0.0 ± 0.0	0.0 ± 0.0	<0.001
	*Pseudomonas*	0.0 ± 0.0	0.0 ± 0.0	0.0 ± 0.0	0.0 ± 0.0	<0.001
	*Sphingobium*	‐	0.0 ± 0.0b	0.4 ± 0.3a	0.1 ± 0.0ab	<0.001
	*Succinivibrio*	0.1 ± 0.1ab	0.1 ± 0.0a	0.0 ± 0.0ab	0.1 ± 0.0a	<0.001
	*Succinivibrionaceae UCG.001*	‐	0.0 ± 0.0	0.0 ± 0.0	0.0 ± 0.0	<0.001
	*Sutterella*	0.7 ± 0.1b	1.5 ± 0.1a	0.9 ± 0.1b	1.4 ± 0.1a	<0.001
Tenericutes	*Anaeroplasma*	0.0 ± 0.0b	0.0 ± 0.0a	0.0 ± 0.0b	0.0 ± 0.0a	<0.001
	*Mycoplasma*	‐	0.0 ± 0.0	0.0 ± 0.0	0.0 ± 0.0	<0.001
	*Clostridia UCG.014 ge*	0.1 ± 0.0	0.1 ± 0.0	0.1 ± 0.0	0.0 ± 0.0	<0.001
Undefined	*Incertae Sedis*	0.0 ± 0.0a	0.0 ± 0.0b	0.0 ± 0.0c	0.0 ± 0.0bc	<0.001
	*Mitochondria ge*	0.0 ± 0.0a	0.0 ± 0.0b	0.0 ± 0.0b	0.0 ± 0.0b	<0.001
	*Oscillospirales ge*	0.0 ± 0.0	0.0 ± 0.0	0.0 ± 0.0	0.0 ± 0.0	<0.001
	*RF39 ge*	‐	0.0 ± 0.0b	0.0 ± 0.0b	0.0 ± 0.0a	<0.001
	*S5.A14a*	‐	0.0 ± 0.0	0.0 ± 0.0	0.0 ± 0.0	<0.001
	*T34 ge*	‐	0.0 ± 0.0	‐	0.0 ± 0.0	<0.001
	*UBA1819*	0.0 ± 0.0a	0.0 ± 0.0b	0.0 ± 0.0b	0.0 ± 0.0a	<0.001
	*UCG.004*	0.0 ± 0.0a	0.0 ± 0.0b	0.0 ± 0.0ab	0.0 ± 0.0ab	<0.001
	*UCG.005*	0.1 ± 0.0b	0.2 ± 0.0a	0.2 ± 0.0a	0.2 ± 0.0a	<0.001
	*UCG.008*	‐	0.0 ± 0.0b	0.0 ± 0.0b	0.0 ± 0.0a	<0.001
	*UCG.009*	0.0 ± 0.0a	0.0 ± 0.0bc	0.0 ± 0.0c	0.0 ± 0.0ab	<0.001
	*Uncultured*	5.9 ± 0.7b	2.9 ± 0.1c	7.8 ± 0.5a	4.0 ± 0.4c	<0.001
	*Uncultured ge*	0.0 ± 0.0c	0.0 ± 0.0b	0.0 ± 0.0c	0.0 ± 0.0a	<0.001
	*X44314*	0.0 ± 0.0bc	0.0 ± 0.0c	0.0 ± 0.0ab	0.0 ± 0.0a	<0.001
	*ZOR0006*	‐	0.0 ± 0.0b	0.0 ± 0.0c	0.0 ± 0.0a	<0.001

*Note*: Seven diets were classed as low‐protein (≤25% DM; *n* = 178 dogs), 17 were moderate‐protein (25%–30% DM; *n* = 208 dogs), 12 were high‐protein (30%–45% DM; *n* = 120 dogs), and three were supra‐protein diets (≥45% DM; *n* = 39 dogs). Results are presented as means with their corresponding SEM. Different letters following the relative abundances denote significant differences (*p* < 0.05). Results marked by a “‐” denote that genera that were not detected in the diet classification. Results denoted as “0.000 ± 0.000” were detected at abundances that round out to 0 at three decimal places but were still detected. Undefined phyla are bacterial genera that have not yet been assigned a taxonomic phylum.

Abbreviations: DM, dry matter; FDR, false discovery rate; SEM, standard error of the mean.

**Figure 4:**
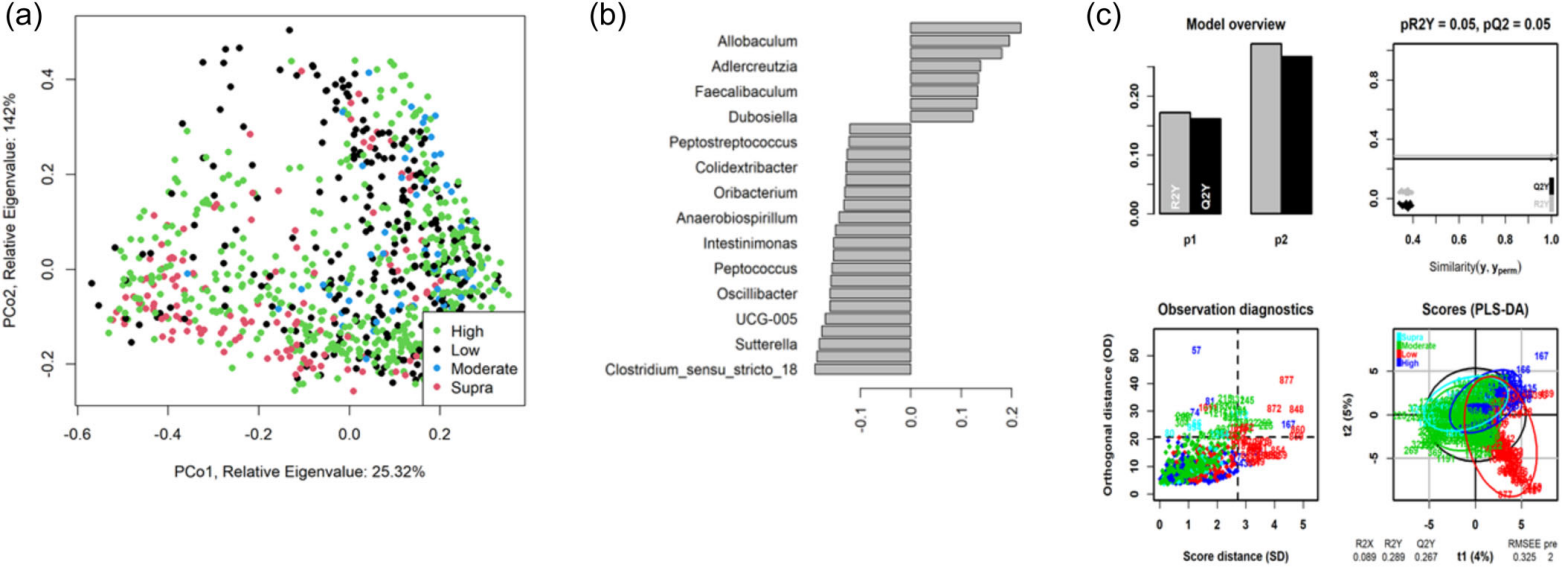
Partial least squares‐discriminant analysis (PLS‐DA) and principal coordinate analysis for dog studies by protein classification. (a) Principal coordinate analysis of Bray–Curtis dissimilarity for all dog protein classifications, *n* = 314, analysis of similarities statistic *R*: 0.1052, significance: 0.001). Each protein classification is presented with a different color. (b) Genera predicted to be most impacted by protein classification, while (c) details the descriptive modeling of the PLS‐DA. A total of seven diets were classed as low‐protein (≤25% DM; *n* = 178 dogs), 17 were moderate‐protein (25%–30% DM; *n* = 208 dogs), 12 were high‐protein (30%–45% DM; *n* = 120 dogs), and three were supra‐protein diets (≥45% DM; *n* = 39 dogs).

As shown in Figure 4, fecal community profiles of the dog vary according to protein content when analyzed using supervised methods. Unsupervised, there seems to be a weak effect (ANOSIM statistic *R*: 0.1052, *p* = 0.001), but PLS‐DA indicated that the relative abundances of *Allobaculum, Adlercreutzia, Faecalibaculum*, and *Duosiella* increase with higher protein content, while genera such as *Peptostreptococcus* and *Colidextribacter* decrease with increasing protein content.

**Figure 5:**
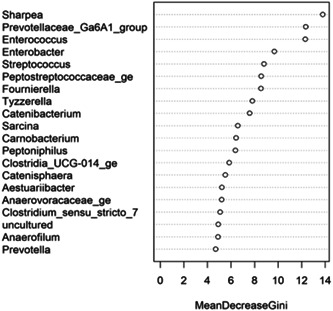
Bacterial genera in the fecal microbiota of the domestic dog associated with driving separations in diversity and richness of the fecal microbiome of dogs assigned to different levels of Protein. A total of seven diets were classed as low‐protein (≤25% DM; *n* = 178 dogs), 17 were moderate‐protein (25%–30% DM; *n* = 208 dogs), 12 were high‐protein (30%–45% DM; *n* = 120 dogs), and three were supra‐protein diets (≥45% DM; *n* = 39 dogs).

The fecal microbiome data were then assessed using random forest analysis to understand which genera were driving separation between the protein classifications. As shown in Figure 5, *Sharpea* was observed to drive the separation of community profiles for the protein classification, followed by *Prevotellaceae Ga6A1 group, Enterococcus*, and *Enterobacter*. All four of these genera were significantly affected by protein classification (*p* < 0.001; Table 3).

Though *Sharpea* was not detected in the low‐protein classification, in all other protein classifications, it had a low relative abundance (0.00 ± 0.00%, 0.05 ± 0.01% and 0.02 ± 0.01% of sequence reads for the moderate‐, high‐, and supra‐protein classifications, respectively). *Prevotellaceae Ga6A1* group had the largest overall relative abundances of these four genera, with relative abundances of 0.57 ± 0.10%, 2.44 ± 0.15%, 1.68 ± 0.19%, and 2.29 ± 0.56% of sequence reads in the low, moderate‐, high‐, and supra‐protein classifications, respectively. *Enterococcus* had the highest relative abundance in the low protein classification, 1.98 ± 0.48% of sequence reads, and decreased through the moderate‐, high‐, and supra‐protein classifications, going from 0.31 ± 0.14% of sequence reads in the moderate‐protein classification to 0.01 ± 0.04% of sequence reads in the high‐protein classification and finally 0.01 ± 0.00% of sequence reads in the supra protein. Meanwhile, *Enterobacter* had a relative abundance of <0.01% of sequence reads in the moderate‐, high‐, and supra‐protein classifications, although in the low protein classification its relative abundance was 0.49 ± 0.14% of sequence reads. The boxplots for the genera most associated with protein level are shown in Figure A4.

### 3.3 Associations between fat level and the fecal microbiome

Of the studies included in this meta‐analysis, 16 were classed as low‐fat (≤15% DM; *n* = 260 dogs), 13 were moderate‐fat (15%–20% DM; *n* = 174 dogs), five were high‐fat (20%–30% DM; *n* = 55 dogs), and five were supra‐fat diets (≥30% DM; *n* = 56 dogs).

**Figure 6:**
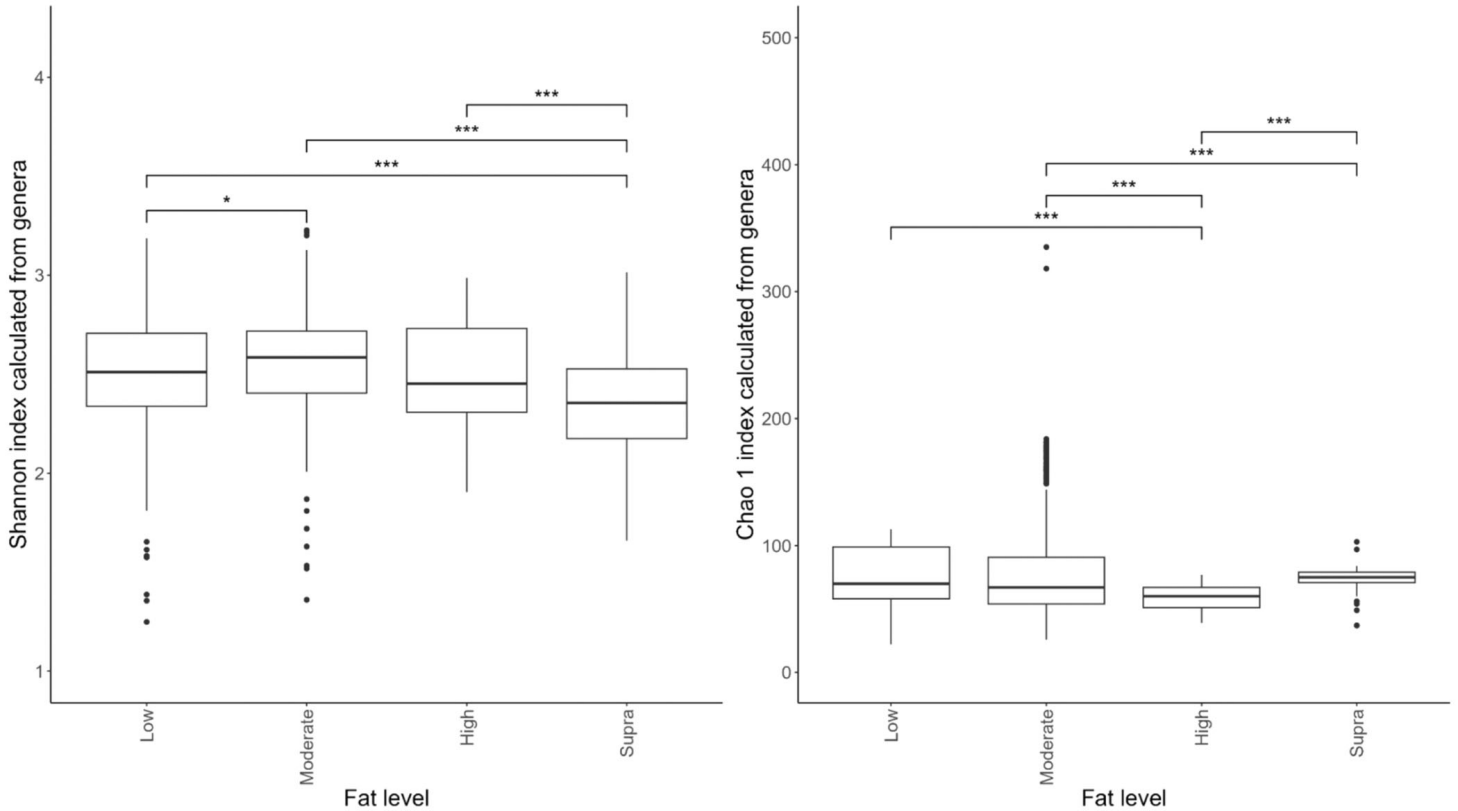
Alpha diversity (Figure 2a) and richness (Figure 2b) of the fecal microbiome of the dog fed differing levels of fat. Sixteen diets were classed as low‐fat (≤15% DM; *n* = 260 dogs), 13 diets were moderate‐fat (15%–20% DM; *n* = 174 dogs), five diets were high‐fat (20%–30% DM; *n* = 55 dogs), and five diets were classed as supra‐fat diets (≥30% DM; *n* = 56 dogs). Statistical differences between fat classifications are denoted by an asterisk (*), where *p < 0.05; ***p < 0.001. Circles denote outliers. Boxes represent the interquartile range between the first and third quartiles. The thick black line inside the box denotes the median.

Despite the overlap between dietary treatment groups, there was a significant effect of diet on the Shannon index (Figure 6a) and Chao 1 indices (Figure 6b) of the fecal microbiome. Alpha diversity was highest in the moderate‐fat diet, whereas bacterial richness was highest in the low‐fat diet.

The effects of dietary fat on the abundance of fecal bacteria genera are shown in Table 4. Of the 226 genera identified in the current study, 222 showed small but statistically different (*p* < 0.05) relative abundances associated with dietary fat content (Table 4). *Fusobacterium* (16.28 ± 0.54% of sequence reads), *Bacteroides* (11.27 ± 0.45% of sequence reads), *Peptoclostridium* (8.23 ± 0.35% of sequence reads), *Blautia* (5.54 ± 0.25% of sequence reads), and *Megamonas* (4.93 ± 0.31% of sequence reads) were the dominant genera observed in dogs assigned to the low‐fat classification (Table 4). *Fusobacterium* (14.66 ± 0.53% of sequence reads), *Bacteroides* (11.51 ± 0.48% of sequence reads), *Prevotella* (11.20 ± 0.63% of sequence reads), *Peptoclostridium* (9.389 ± 0.48% of sequence reads), and *Alloprevotella* (5.19 ± 0.23% of sequence reads) were the most abundant genera observed in the fecal microbiome of dogs assigned to the moderate‐fat classification.

**Table 4:** Relative abundances (% of sequence reads) averaging above 0.001% of sequence reads in one treatment of the fecal microbiome of the dog fed differing levels of fat.

	Genus	Low fat, mean ± SEM	Moderate fat, mean ± SEM	High fat, mean ± SEM	Supra fat, mean ± SEM	FDR‐adjusted, *p* value
Actinobacteria	*Actinomyces*	‐	0.0 ± 0.0	0.0 ± 0.0	‐	<0.001
	*Atopobiaceae_ge*	0.0 ± 0.0b	0.0 ± 0.0ab	‐	0.0 ± 0.0a	<0.001
	*Bifidobacterium*	‐	0.0 ± 0.0b	‐	0.1 ± 0.1a	<0.001
	*Collinsella*	‐	0.0 ± 0.0b	0.0 ± 0.0a	‐	<0.001
	*Coriobacteriaceae UCG.002*	0.0 ± 0.0b	0.0 ± 0.0b	0.0 ± 0.0b	0.0 ± 0.0a	<0.001
	*Coriobacterium*	‐	0.0 ± 0.0	‐	‐	<0.001
	*Corynebacterium*	0.0 ± 0.0a	0.0 ± 0.0b	‐	‐	<0.001
	*Cutibacterium*	‐	0.0 ± 0.0	‐	‐	<0.001
	*Denitrobacterium*	0.0 ± 0.0	0.0 ± 0.0	‐	0.0 ± 0.0	<0.001
	*Enorma*	0.0 ± 0.0ab	0.0 ± 0.0a	0.0 ± 0.0c	0.0 ± 0.0bc	<0.001
	*Libanicoccus*	1.6 ± 0.2c	2.7 ± 0.3b	1.9 ± 0.2bc	6.7 ± 0.7a	<0.001
	*Olsenella*	3.4 ± 0.2b	5.2 ± 0.2a	1.8 ± 0.3c	3.5 ± 0.5b	<0.001
	*Parvibacter*	‐	0.0 ± 0.0b	‐	0.0 ± 0.0a	<0.001
	*Pseudarthrobacter*	1.1 ± 0.1a	1.2 ± 0.1a	0.7 ± 0.1b	0.9 ± 0.1ab	<0.001
	*Senegalimassilia*	0.0 ± 0.0a	0.0 ± 0.0b	0.0 ± 0.0b	0.0 ± 0.0b	<0.001
	*Slackia*	‐	0.1 ± 0.0a	‐	0.0 ± 0.0b	<0.001
	*Tetrasphaera*	0.0 ± 0.0b	0.0 ± 0.0a	0.0 ± 0.0bc	0.0 ± 0.0c	<0.001
	*Trueperella*	‐	0.0 ± 0.0	‐	‐	<0.001
	*Alistipes*	0.0 ± 0.0b	0.0 ± 0.0a	‐	‐	<0.001
Bacteroidetes	*Alloprevotella*	0.0 ± 0.0b	0.0 ± 0.0a	‐	‐	<0.001
	*Bacteroides*	‐	0.0 ± 0.0b	0.0 ± 0.0a	‐	<0.001
	*Barnesiella*	0.0 ± 0.0b	0.0 ± 0.0a	0.0 ± 0.0ab	0.0 ± 0.0ab	<0.001
	*Butyricimonas*	0.4 ± 0.0b	0.3 ± 0.0c	0.6 ± 0.1a	0.3 ± 0.0bc	<0.001
	*Muribaculaceae ge*	‐	0.0 ± 0.0b	‐	0.0 ± 0.0a	<0.001
	*Odoribacter*	0.0 ± 0.0	0.0 ± 0.0	‐	‐	<0.001
	*Parabacteroides*	0.0 ± 0.0	0.0 ± 0.0	‐	‐	<0.001
	*Paraprevotella*	11.3 ± 0.5a	11.5 ± 0.5a	7.0 ± 1.1b	10.0 ± 0.6a	<0.001
	*Porphyromonas*	‐	0.0 ± 0.0	‐	‐	<0.001
	*Prevotella*	1.1 ± 0.2a	0.2 ± 0.0b	0.1 ± 0.0b	0.6 ± 0.1b	<0.001
	*Prevotellaceae NK3B31 group*	5.5 ± 0.2b	4.5 ± 0.2c	14.6 ± 1.2a	3.7 ± 0.3c	<0.001
	*Prevotellaceae UCG.001*	‐	0.0 ± 0.0b	‐	0.0 ± 0.0a	<0.001
	*Prevotellaceae UCG.003*	0.1 ± 0.0a	0.1 ± 0.0b	0.1 ± 0.0ab	0.0 ± 0.0c	<0.001
Deferribacteres	*Mucispirillum*	0.0 ± 0.0	0.0 ± 0.0	‐	0.0 ± 0.0	<0.001
Euryarchaeota	*Methanobrevibacter*	0.0 ± 0.0ab	0.0 ± 0.0a	‐	0.0 ± 0.0b	<0.001
	*Methanosphaera*	‐	0.0 ± 0.0	‐	0.0 ± 0.0	<0.001
Firmicutes	*Acetanaerobacterium*	0.0 ± 0.0d	0.0 ± 0.0c	0.1 ± 0.0a	0.1 ± 0.0b	<0.001
	*Agathobacter*	0.0 ± 0.0b	0.0 ± 0.0b	0.1 ± 0.0a	‐	<0.001
	*Allisonella*	‐	0.0 ± 0.0	‐	‐	<0.001
	*Allobaculum*	0.7 ± 0.1b	1.0 ± 0.1b	2.1 ± 0.4a	0.5 ± 0.4b	<0.001
	*Amnipila*	0.0 ± 0.0c	0.1 ± 0.0bc	0.5 ± 0.1a	0.1 ± 0.0b	<0.001
	*Anaerofilum*	0.0 ± 0.0b	0.1 ± 0.0a	0.0 ± 0.0b	0.1 ± 0.0a	<0.001
	*Anaerofustis*	0.3 ± 0.1b	0.3 ± 0.1b	‐	0.9 ± 0.2a	<0.001
	*Anaerospora*	0.0 ± 0.0a	0.0 ± 0.0b	‐	‐	<0.001
	*Anaerostignum*	‐	0.0 ± 0.0a	‐	0.0 ± 0.0b	<0.001
	*Anaerostipes*	0.0 ± 0.0	0.0 ± 0.0	‐	‐	<0.001
	*Anaerotruncus*	0.0 ± 0.0a	0.0 ± 0.0a	‐	0.0 ± 0.0b	<0.001
	*Anaerovibrio*	0.0 ± 0.0a	0.0 ± 0.0b	‐	‐	<0.001
	*Anaerovoracaceae ge*	0.1 ± 0.0a	0.0 ± 0.0b	0.0 ± 0.0b	0.1 ± 0.1ab	<0.001
	*Angelakisella*	0.0 ± 0.0b	0.0 ± 0.0a	0.0 ± 0.0b	0.0 ± 0.0b	<0.001
	*Bacillus*	2.3 ± 0.2b	1.3 ± 0.2c	4.4 ± 0.7a	2.0 ± 0.2b	<0.001
	*Blautia*	0.1 ± 0.0a	0.0 ± 0.0b	‐	‐	<0.001
	*Butyricicoccus*	0.0 ± 0.0	0.0 ± 0.0	‐	‐	<0.001
	*Candidatus Arthromitus*	0.0 ± 0.0a	0.0 ± 0.0b	‐	‐	<0.001
	*Candidatus Soleaferrea*	0.2 ± 0.1a	0.0 ± 0.0b	‐	‐	<0.001
	*Candidatus Stoquefichus*	0.0 ± 0.0a	0.0 ± 0.0a	0.0 ± 0.0b	0.0 ± 0.0b	<0.001
	*Caproiciproducens*	1.7 ± 0.1b	0.8 ± 0.1c	2.9 ± 0.3a	0.7 ± 0.1c	<0.001
	*Carnobacterium*	‐	0.0 ± 0.0	‐	‐	<0.001
	*Catellicoccus*	0.0 ± 0.0b	0.0 ± 0.0a	0.0 ± 0.0a	0.0 ± 0.0b	<0.001
	*Catenibacterium*	0.0 ± 0.0b	0.0 ± 0.0b	‐	0.1 ± 0.0a	<0.001
	*Cellulosilyticum*	0.0 ± 0.0b	0.0 ± 0.0b	‐	0.0 ± 0.0a	<0.001
	*CHKCI001*	0.0 ± 0.0b	0.0 ± 0.0a	‐	‐	<0.001
	*Clostridioides*	0.0 ± 0.0	0.0 ± 0.0	0.0 ± 0.0	‐	<0.001
	*Clostridium sensu stricto* 1	0.0 ± 0.0a	0.0 ± 0.0b	‐	‐	<0.001
	*Clostridium sensu stricto* 13	0.0 ± 0.0ab	0.0 ± 0.0b	‐	0.0 ± 0.0a	<0.001
	*Clostridium sensu stricto* 2	‐	0.0 ± 0.0a	0.0 ± 0.0b	‐	<0.001
	*Colidextribacter*	0.1 ± 0.0b	0.4 ± 0.1a	0.1 ± 0.0b	0.5 ± 0.1a	<0.001
	*Coprococcus*	0.2 ± 0.0a	0.0 ± 0.0b	0.0 ± 0.0b	0.0 ± 0.0b	<0.001
	*Defluviitaleaceae UCG.011*	0.6 ± 0.2	0.4 ± 0.2	0.1 ± 0.1	‐	<0.001
	*Dialister*	0.1 ± 0.0a	0.0 ± 0.0b	‐	0.0 ± 0.0b	<0.001
	*Dorea*	0.0 ± 0.0b	0.0 ± 0.0a	0.0 ± 0.0a	‐	<0.001
	*Dubosiella*	0.1 ± 0.0c	0.2 ± 0.0b	0.5 ± 0.1a	0.1 ± 0.0c	<0.001
	*Enterococcus*	0.0 ± 0.0b	0.0 ± 0.0a	0.0 ± 0.0a	‐	<0.001
	*Epulopiscium*	0.2 ± 0.0a	0.2 ± 0.0a	0.3 ± 0.1a	0.0 ± 0.0b	<0.001
	*Erysipelatoclostridium*	2.8 ± 0.3a	0.4 ± 0.1b	1.0 ± 0.4b	0.3 ± 0.1b	<0.001
	*Erysipelotrichaceae ge*	0.0 ± 0.0	0.0 ± 0.0	0.0 ± 0.0	0.0 ± 0.0	<0.001
	*Erysipelotrichaceae UCG.003*	2.2 ± 0.2a	2.2 ± 0.1a	1.2 ± 0.2b	2.4 ± 0.2a	<0.001
	*Eubacterium*	0.0 ± 0.0b	0.1 ± 0.0a	0.0 ± 0.0b	0.1 ± 0.0a	<0.001
	*Faecalibaculum*	0.0 ± 0.0	0.0 ± 0.0	0.0 ± 0.0	0.1 ± 0.0	<0.001
	*Faecalicoccus*	0.0 ± 0.0b	0.0 ± 0.0ab	‐	0.0 ± 0.0a	<0.001
	*Family XIII AD3011 group*	0.0 ± 0.0	0.0 ± 0.0	‐	‐	<0.001
	*Family XIII UCG.001*	0.1 ± 0.0b	0.2 ± 0.0a	0.1 ± 0.0b	0.1 ± 0.0b	<0.001
	*Flavonifractor*	0.0 ± 0.0b	0.0 ± 0.0b	0.0 ± 0.0a	‐	<0.001
	*Fournierella*	0.0 ± 0.0	0.0 ± 0.0	‐	‐	<0.001
	*Fusibacter*	16.3 ± 0.5b	14.7 ± 0.5b	9.2 ± 1.1c	22.8 ± 1.1a	<0.001
	*GCA.900066575*	0.0 ± 0.0c	0.1 ± 0.0b	0.3 ± 0.0a	0.0 ± 0.0c	<0.001
	*Hathewaya*	0.0 ± 0.0a	0.0 ± 0.0b	0.0 ± 0.0ab	‐	<0.001
	*Holdemanella*	0.0 ± 0.0a	0.0 ± 0.0b	‐	‐	<0.001
	*Holdemania*	0.1 ± 0.0	0.1 ± 0.0	0.0 ± 0.0	0.1 ± 0.0	<0.001
	*Howardella*	‐	‐	‐	0.1 ± 0.1	<0.001
	*Hungateiclostridium*	0.5 ± 0.1c	0.9 ± 0.1b	1.3 ± 0.3a	0.2 ± 0.0d	<0.001
	*Hydrogenoanaerobacterium*	0.0 ± 0.0ab	0.0 ± 0.0b	0.0 ± 0.0a	0.0 ± 0.0c	<0.001
	*Intestinibacter*	0.0 ± 0.0a	0.0 ± 0.0b	‐	0.0 ± 0.0a	<0.001
	*Lachnoclostridium*	0.0 ± 0.0c	0.3 ± 0.1b	‐	0.6 ± 0.1a	<0.001
	*Lachnospira*	0.0 ± 0.0a	0.0 ± 0.0b	0.0 ± 0.0a	0.0 ± 0.0b	<0.001
	*Lachnospiraceae AC2044 group*	0.0 ± 0.0c	0.1 ± 0.0b	0.4 ± 0.0a	0.0 ± 0.0c	<0.001
	*Lachnospiraceae FCS020 group*	0.0 ± 0.0a	0.0 ± 0.0b	‐	0.0 ± 0.0c	<0.001
	*Lachnospiraceae ge*	0.0 ± 0.0	0.0 ± 0.0	0.0 ± 0.0	‐	<0.001
	*Lachnospiraceae NC2004 group*	0.7 ± 0.1b	0.5 ± 0bc	1.1 ± 0.1a	0.4 ± 0.0c	<0.001
	*Lachnospiraceae ND3007 group*	0.2 ± 0.0a	0.1 ± 0.0b	0.1 ± 0.0b	0.1 ± 0.0b	<0.001
	*Lachnospiraceae NK3A20 group*	‐	0.0 ± 0.0a	0.0 ± 0.0b	0.0 ± 0.0b	<0.001
	*Lachnospiraceae NK4A136 group*	0.0 ± 0.0b	0.0 ± 0.0b	0.0 ± 0.0a	‐	<0.001
	*Lachnospiraceae UCG.003*	3.7 ± 0.2b	1.7 ± 0.1d	4.7 ± 0.4a	2.5 ± 0.2c	<0.001
	*Lachnospiraceae UCG.010*	‐	0.0 ± 0.0a	‐	0.0 ± 0.0a	<0.001
	*Lachnospiraceae XPB1014 group*	‐	0.0 ± 0.0	‐	‐	<0.001
	*Lactobacillus*	0.2 ± 0.0a	0.3 ± 0.0a	0.2 ± 0.0b	0.1 ± 0.0b	<0.001
	*Lactococcus*	‐	0.0 ± 0.0b	0.0 ± 0.0a	‐	<0.001
	*Leuconostoc*	‐	0.0 ± 0.0a	0.0 ± 0.0a	0.0 ± 0.0b	<0.001
	*Megamonas*	0.0 ± 0.0b	0.0 ± 0.0a	0.0 ± 0.0b	0.0 ± 0.0b	<0.001
	*Megasphaera*	1.8 ± 0.3b	4.9 ± 0.5a	5.3 ± 1.1a	4.0 ± 0.6a	<0.001
	*Mitsuokella*	0.0 ± 0.0b	0.0 ± 0.0b	0.1 ± 0.0a	‐	0.005
	*Monoglobus*	0.0 ± 0.0b	0.0 ± 0.0c	0.0 ± 0.0a	‐	<0.001
	*Negativibacillus*	‐	0.0 ± 0.0	‐	‐	<0.001
	*Oribacterium*	0.3 ± 0.1a	0.0 ± 0.0b	0.0 ± 0.0b	0.1 ± 0.0b	<0.001
	*Oscillibacter*	4.9 ± 0.3a	2.3 ± 0.2b	2.3 ± 0.6b	0.3 ± 0.1c	<0.001
	*Oscillospira*	0.0 ± 0.0b	0.1 ± 0.0ab	‐	0.1 ± 0.1a	<0.001
	*Oscillospiraceae ge*	0.0 ± 0.0b	‐	‐	0.0 ± 0.0a	<0.001
	*Paeniclostridium*	0.0 ± 0.0b	0.0 ± 0.0b	‐	0.2 ± 0.0a	<0.001
	*Paludicola*	0.0 ± 0.0	0.0 ± 0.0	‐	‐	<0.001
	*Papillibacter*	‐	0.0 ± 0.0b	‐	0.0 ± 0.0a	<0.001
	*Peptoclostridium*	0.0 ± 0.0a	0.0 ± 0.0b	‐	‐	<0.001
	*Peptococcus*	0.0 ± 0.0ab	0.0 ± 0.0a	0.0 ± 0.0b	0.0 ± 0.0b	<0.001
	*Peptoniphilus*	0.2 ± 0.0c	1.6 ± 0.2b	0.2 ± 0.1c	3.3 ± 0.5a	<0.001
	*Peptostreptococcaceae ge*	‐	0.0 ± 0.0b	‐	0.0 ± 0.0a	<0.001
	*Peptostreptococcus*	‐	‐	‐	0.0 ± 0.0	<0.001
	*Phascolarctobacterium*	0.2 ± 0.0a	0.2 ± 0.0b	0.1 ± 0.0c	0.1 ± 0.0c	<0.001
	*Phocea*	0.0 ± 0.0b	0.0 ± 0.0a	‐	0.0 ± 0.0a	<0.001
	*Pseudoflavonifractor*	‐	0.0 ± 0.0b	‐	0.1 ± 0.1a	0.098
	*Pygmaiobacter*	0.0 ± 0.0b	0.0 ± 0.0a	‐	0.0 ± 0.0c	<0.001
	*Robinsoniella*	0.1 ± 0.0a	0.0 ± 0.0b	‐	0.0 ± 0.0b	<0.001
	*Romboutsia*	0.0 ± 0.0a	0.0 ± 0.0ab	0.0 ± 0.0c	0.0 ± 0.0b	<0.001
	*Roseburia*	0.0 ± 0.0b	0.0 ± 0.0a	0.0 ± 0.0c	0.0 ± 0.0c	<0.001
	*Ruminococcus*	0.0 ± 0.0a	0.0 ± 0.0b	‐	0.0 ± 0.0b	<0.001
	*Sellimonas*	0.0 ± 0.0	0.0 ± 0.0	‐	0.0 ± 0.0	<0.001
	*Sharpea*	0.0 ± 0.0b	0.0 ± 0.0b	0.0 ± 0.0a	‐	<0.001
	*Shuttleworthia*	0.0 ± 0.0b	0.0 ± 0.0a	‐	‐	<0.001
	*Sporosarcina*	0.1 ± 0.0b	0.2 ± 0.0a	0.0 ± 0.0c	0.2 ± 0.0a	<0.001
	*Staphylococcus*	0.1 ± 0.0b	0.2 ± 0.0b	1.1 ± 0.5a	0.2 ± 0.0b	<0.001
	*Streptococcus*	0.0 ± 0.0a	0.0 ± 0.0a	‐	0.0 ± 0.0b	<0.001
	*Subdoligranulum*	1.1 ± 0.1c	1.5 ± 0.1b	0.4 ± 0.1d	2.1 ± 0.2a	<0.001
	*Turicibacter*	8.2 ± 0.4b	9.4 ± 0.5b	15.8 ± 1.3a	9.4 ± 0.8b	<0.001
	*Tuzzerella*	0.8 ± 0.1a	0.4 ± 0.0c	0.7 ± 0.1ab	0.5 ± 0.1bc	<0.001
	*Tyzzerella*	0.7 ± 0.1a	0.0 ± 0.0b	‐	‐	<0.001
	*Vagococcus*	0.0 ± 0.0a	0.0 ± 0.0a	‐	0.0 ± 0.0b	<0.001
	*Weissella*	1.1 ± 0.2a	0.4 ± 0.1b	0.1 ± 0.1b	0.2 ± 0.0b	<0.001
Fusobacteria	*Cetobacterium*	1.8 ± 0.1a	1.3 ± 0.1b	1.1 ± 0.2bc	0.6 ± 0.1c	<0.001
	*Fusobacterium*	0.0 ± 0.0a	0.0 ± 0.0a	‐	0.0 ± 0.0b	<0.001
	*Oceanivirga*	0.1 ± 0.0	0.0 ± 0.0	‐	‐	<0.001
	*Streptobacillus*	‐	0.0 ± 0.0	‐	0.0 ± 0.0	<0.001
Lentisphaerae	*Victivallis*	4.7 ± 0.5b	11.2 ± 0.6a	4.7 ± 0.8b	1.2 ± 0.3c	0.076
Proteobacteria	*Acinetobacter*	2.4 ± 0.2a	1.6 ± 0.1b	0.5 ± 0.1c	2.0 ± 0.4ab	<0.001
	*Actinobacillus*	‐	0.0 ± 0.0b	‐	0.0 ± 0.0a	<0.001
	*Aeromonas*	‐	0.0 ± 0.0a	‐	0.0 ± 0.0b	<0.001
	*Aestuariibacter*	0.0 ± 0.0b	0.0 ± 0.0b	‐	0.1 ± 0.0a	<0.001
	*Anaerobiospirillum*	0.0 ± 0.0b	0.0 ± 0.0c	‐	0.0 ± 0.0a	0.547
	*Bilophila*	‐	0.0 ± 0.0	‐	‐	0.098
	*Bosea*	0.0 ± 0.0a	0.0 ± 0.0b	‐	0.0 ± 0.0ab	<0.001
	*Campylobacter*	0.2 ± 0.0a	0.0 ± 0.0bc	‐	0.1 ± 0.0b	<0.001
	*Citrobacter*	‐	0.0 ± 0.0b	‐	0.0 ± 0.0a	<0.001
	*Cupriavidus*	0.0 ± 0.0c	0.1 ± 0.0a	0.0 ± 0.0c	0.1 ± 0.0b	<0.001
	*Desulfovibrio*	0.0 ± 0.0a	0.0 ± 0.0b	‐	‐	<0.001
	*Enterobacter*	0.8 ± 0.1b	0.3 ± 0.0c	0.3 ± 0.1c	1.4 ± 0.1a	<0.001
	*Escherichia.Shigella*	0.0 ± 0.0a	0.0 ± 0.0a	0.0 ± 0.0a	0.0 ± 0.0a	<0.001
	*Hafnia.Obesumbacterium*	0.0 ± 0.0	0.0 ± 0.0	‐	0.0 ± 0.0a	<0.001
	*Helicobacter*	0.0 ± 0.0b	0.0 ± 0.0c	‐	0.1 ± 0.0a	<0.001
	*Histophilus*	0.2 ± 0.0b	0.4 ± 0.2a	0.0 ± 0.0b	‐	<0.001
	*Parasutterella*	‐	0.0 ± 0.0b	‐	0.1 ± 0.0a	<0.001
	*Plesiomonas*	‐	0.0 ± 0.0a	‐	0.0 ± 0.0b	<0.001
	*Pseudomonas*	0.2 ± 0.0b	0.1 ± 0.0c	0.3 ± 0.0a	0.0 ± 0.0d	<0.001
	*Sphingobium*	‐	0.0 ± 0.0b	0.0 ± 0.0a	‐	<0.001
	*Succinivibrio*	‐	0.0 ± 0.0b	‐	0.7 ± 0.4a	<0.001
	*Succinivibrionaceae UCG.001*	0.0 ± 0.0a	0.0 ± 0.0b	‐	‐	<0.001
	*Sutterella*	0.0 ± 0.0b	0.0 ± 0.0b	‐	0.0 ± 0.0a	<0.001
Spirochaetes	*Leptospira*	‐	0.0 ± 0.0b	‐	0.2 ± 0.2a	0.002
Tenericutes	*Anaeroplasma*	2.6 ± 0.4a	2.7 ± 0.4a	3.1 ± 0.6a	0.1 ± 0.0b	<0.001
	*Mycoplasma*	0.0 ± 0.0	0.0 ± 0.0	‐	0.0 ± 0.0	<0.001
Undefined	*Chloroplast_ge*	0.1 ± 0.0b	0.2 ± 0.0a	0.0 ± 0.0b	0.0 ± 0.0b	<0.001
	*Clostridia UCG.014 ge*	0.0 ± 0.0b	0.0 ± 0.0b	0.0 ± 0.0b	0.1 ± 0.1a	<0.001
	*Dojkabacteria ge*	1.3 ± 0.1a	1.4 ± 0.1a	0.4 ± 0.1c	0.8 ± 0.1b	0.002
	*DTU089*	0.0 ± 0.0b	0.0 ± 0.0a	‐	‐	<0.001
	*Erysipelatoclostridiaceae ge*	0.4 ± 0.1a	0.1 ± 0.0b	0.2 ± 0.1ab	0.3 ± 0.0ab	<0.001
	*Gastranaerophilales ge*	0.0 ± 0.0a	0.0 ± 0.0b	‐	‐	<0.001
	*Incertae Sedis*	‐	0.0 ± 0.0b	‐	0.1 ± 0.1a	<0.001
	*Mitochondria ge*	1.6 ± 0.2a	1.9 ± 0.2a	1.2 ± 0.3ab	0.8 ± 0.2b	<0.001
	*Oscillospirales ge*	0.0 ± 0.0b	0.0 ± 0.0a	0.0 ± 0.0ab	‐	<0.001
	*RF39 ge*	0.2 ± 0.0a	0.1 ± 0.0b	0.1 ± 0.0ab	0.0 ± 0.0b	<0.001
	*S5.A14a*	0.0 ± 0.0	0.0 ± 0.0	‐	‐	<0.001
	*UBA1819*	0.0 ± 0.0ab	0.0 ± 0.0a	‐	0.0 ± 0.0b	<0.001
	*UCG.003*	0.2 ± 0.0	0.2 ± 0.0	0.2 ± 0.0	0.2 ± 0.0	<0.001
	*UCG.004*	‐	0.0 ± 0.0	‐	0.0 ± 0.0	<0.001
	*UCG.008*	4.3 ± 0.3b	3.6 ± 0.2b	3.9 ± 0.4b	9.1 ± 0.6a	<0.001
	*UCG.009*	0.0 ± 0.0b	0.0 ± 0.0a	0.0 ± 0.0b	0.0 ± 0.0b	<0.001
	*Uncultured*	0.0 ± 0.0a	0.0 ± 0.0b	‐	‐	<0.001
	*X44314*	0.0 ± 0.0	0.0 ± 0.0	0.0 ± 0.0	‐	<0.001
	*ZOR0006*	‐	0.0 ± 0.0	‐	‐	<0.001

*Note*: Sixteen diets were classed as low‐fat (≤15% DM; *n* = 260 dogs), 13 diets were moderate‐fat (15%–20% DM; *n* = 174 dogs), five diets were high‐fat (20%–30% DM; *n* = 55 dogs), and five diets were classed as supra‐fat diets (≥30% DM; *n* = 56 dogs). Results are presented as means with their corresponding SEM. Different letters following the relative abundances denote significant differences (*p* < 0.05). Results marked by a “‐” denote that the genera were not detected in this diet classification. Results denoted as “0.000 ± 0.000” were detected at abundances that round out to 0 at three decimal places, but were still detected. Undefined phyla are bacterial genera that have not yet been assigned a taxonomic phyla that they belong to.

Abbreviations: DM, dry matter; FDR, false discovery rate; SEM, standard error of the mean.

In the fecal microbiome of dogs assigned to the high‐fat classification, the dominant genera were *Peptoclostridium* (15.76 ± 1.32% of sequence reads), *Blautia* (14.61 ± 1.24% of sequence reads), *Fusobacterium* (9.16 ± 1.12% of sequence reads), *Bacteroides* (7.01 ± 1.14% of sequence reads), and *Lactobacillus* (5.31 ± 1.14% of sequence reads). Dominant bacterial genera observed in the fecal microbiome of dogs assigned to the supra‐fat diet were *Fusobacterium* (22.76 ± 1.10% of sequence reads), *Bacteroides* (10.01 ± 0.64% of sequence reads), *Peptoclostridium* (9.40 ± 0.82% of sequence reads), uncultured bacteria (9.08 ± 0.64% of sequence reads), and *Allobacullum* (6.68 ± 0.69% of sequence reads).

**Figure 7:**
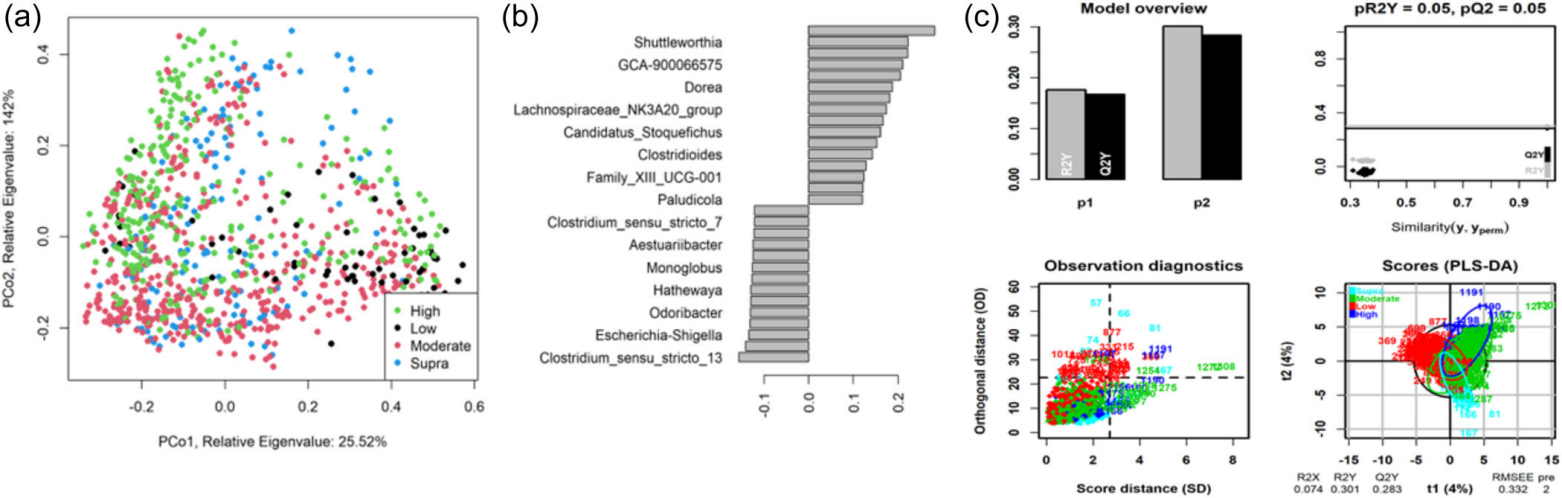
Partial least squares‐discriminant analysis (PLS‐DA) and principal coordinate analysis for dog studies by fat classification. (a) details principal coordinate analysis of Bray–Curtis dissimilarity for all dog fat classifications, *n* = 314, analysis of similarities statistic *R*: 0.1179, significance: 0.001). Each fat classification is presented with a different color. (b) details the genera predicted to be most impacted by fat classification, while (c) details the descriptive modeling of the PLS‐DA. A total of 16 diets were classed as low‐fat (≤15% DM; *n* ****= 260 dogs), 13 diets were moderate‐fat (15%–20% DM; *n* ****= 174 dogs), five diets were high‐fat (20%–30% DM; *n* ****= 55 dogs), and five diets were classed as supra‐fat diets (≥30% DM; *n* ****= 56 dogs).

As shown in Figure 7a, the fecal microbiome of dogs showed limited separation (ANOSIM statistic *R*: 0.1179, *p* = 0.001) based on fat classifications. PLS‐DA (Figure 7b,c) indicated that the relative abundance of *Shuttleworthia, Dorea*, and *Clostridiodes* increased relative to higher fat content. In contrast, genera such as *Aestuariibacter* and *Monoglobus* decreased with increasing fat levels.

**Figure 8:**
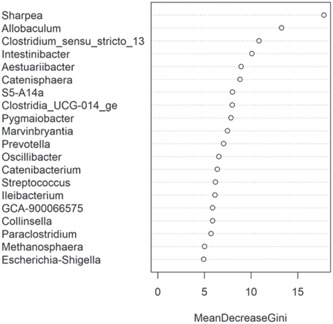
Bacterial genera in the fecal microbiota of the domestic dog associated with driving separations in diversity and richness of the fecal microbiome when assessed by fat classifications. A total of 16 diets were classed as low‐fat (≤15% dry matter [DM]; *n* = 260 dogs), 13 diets were moderate‐fat (15%–20% DM; *n* = 174 dogs), five diets were high‐fat (20%–30% DM; *n* = 55 dogs), and five diets were classed as supra‐fat diets (≥30% DM; *n* = 56 dogs).

The fecal microbiome was then assessed to understand what genera were driving the separation between the fat classifications. As shown in Figure 8, *Sharpea* was observed to drive the separation of community profiles for the fat classification, followed by *Allobaculum, Clostridium sensu stricto 13*, and *Intestnibacter*. All four of these genera were significantly affected by fat classification (*p* < 0.001; Table 4). The boxplots for the genera most associated with fat level are shown in Figure A4.

## 4 DISCUSSION

This meta‐analysis aimed to understand the impact of increasing dietary protein or fat levels on the composition of the fecal microbiome of the dog and indicated that this is affected by both dietary crude protein and crude fat levels. This analysis showed the separation of the fecal microbial communities according to dietary protein and, to a lesser extent, dietary fat levels. Corresponding analysis of this indicated that, despite its relatively low abundance across all dietary classifications, *Sharpea* was responsible for driving this separation in both instances.

The relative abundance of *Sharpea* was significantly affected by both dietary protein and fat levels, with the largest relative abundances observed in the high‐protein and supra‐fat classifications. However, overall *Sharpea* was present in relatively low abundances (<0.1% of sequence reads). *Sharpea* was only present in 4 studies after filtering, although three of these studies were the three studies containing the “supra” protein levels and the other study had a large number of “high” protein samples further highlighting the association of *Sharpea* with higher protein levels. *Sharpea* is a lactate and acetate producer in ruminants (Kumar et al., 2018), and its relative abundance has been observed to decrease in piglets with diarrhea (Yang et al., 2017). However in the dog, the role of *Sharpea* is unknown, possibly owing to its low relative abundance; in fact, *Sharpea* has only been reported in one publication that assessed the fecal microbiota of dogs (Sturgeon, 2014). Though there is an increasing interest in the functionality of the relatively low‐abundant constituents of the human microbiome (Cena et al., 2021), this is still an unexplored field in the dog. This highlights the potential of techniques such as meta‐analysis in increasing our understanding of the role of relatively low‐abundant genera in the dog.

Aside from *Sharpea*, *Prevotellaceae Ga6A1 group* (observed range across diet classifications 0.6%–2.4% sequence reads) and *Enterococcus* (observed range across diet classifications 0%–2.0% sequence reads) appear to be pivotal in driving the differences observed in the microbial profiles between dietary protein classifications in healthy dogs; again, these genera were observed to have a relatively low abundance in the fecal microbiome of the dog. In the dog, *Prevotellaceae Ga6A1 group* relative abundances decreased in association with weight loss in overweight dogs fed high‐protein diets (Phungviwatnikul et al., 2021). In mice, it was found that probiotic dietary application of *Bifidobacterium* resulted in decreases in the *Prevotellaceae Ga6A1 group* (Gryaznova et al., 2022). Interestingly, in the high‐protein dog diet, *Bifidobacterium* relative abundances increased as the *Prevotellacaea Ga6A1 group* decreased, although this possible correlation was not investigated further (Phungviwatnikul et al., 2021). This suggests, though tenuously, that the acetate and succinate production from the *Prevotellaceae Ga6A1 group* is not as useful as the lactate production from *Bifidobacterium*. However, further research is needed in this area to understand this relationship further.

The relative abundance of *Enterococcus* decreased as dietary protein content increased, consistent with previously reported results (Phungviwatnikul et al., 2021; Pinna et al., 2016). *Enterococcus* spp., have been used as probiotics in dogs (Hanifeh et al., 2021; Pilla et al., 2019; Schmitz, Glanemann, et al., 2015; Schmitz, Werling, et al., 2015; Strompfová et al., 2004) and are primarily acetate producers (Wu et al., 2021). In addition, enterococci also produce bile salt hydrolases (Phungviwatnikul et al., 2021), which catalyze the deconjugation of glycol‐ and tauro‐conjugated bile acids, thereby undertaking a role in fat metabolism (Ren et al., 2011; Xu et al., 2019). In this analysis, the relative abundance of *Enterococcus* decreased as dietary fat content increased. The results obtained here suggest that in healthy dogs *Enterococcus* may perform an important role in instances of low protein and/or fat.

In terms of response to dietary fat, *Allobaculum* (observed range across diet classifications 0.5%–2.1% sequence reads) and *Clostridium sensu stricto 13* (observed range across diet classifications 0.00%–0.01% sequence reads) were also important for driving separation between community profiles in the healthy dog. *Allobaculum* had the highest relative abundance (0.50 ± 0.36% of sequence reads) in the supra‐fat classification, which had an average CHO content of 7.95% DM (c. 6% of sequence reads). The relative abundance of *Allobaculum* has been found to decrease in the feces of healthy dogs fed diets free of animal protein but containing animal fats (compared to baseline) (Bresciani et al., 2018). However, in this study, we did not separate results based on protein type. Other studies have observed a decrease in the relative abundance of *Allobaculum* in supra‐protein/moderate‐fat diets, compared to extruded kibble diets, which based on the classifications in this study would be supra‐protein/moderate‐fat compared to moderate‐protein/high‐fat diets (Bermingham et al., 2017). Additionally, other publications show conflicting changes in *Allobaculum* relative abundance relating to weight loss or obesity in the dog. For example, Phungviwatnikul et al. (2021). observed an increase in the relative abundance of *Allobaculum* in overweight dogs compared to healthy‐weight dogs fed high‐protein (30%–45% DM) and high‐fat (20%–30% DM) diets. In contrast, Macedo et al. (2022). observed a decrease in the relative abundance of *Allobaculum* in overweight dogs compared to healthy weight dogs fed a moderate‐protein (25%–30% DM)/low‐fat diet (5%–15% DM). As *Allobaculum* hydrolyzes monodisaccharides and disaccharides rather than starch (Greetham et al., 2004), diets high in starch or low in monosaccharides and disaccharides—or both—may be responsible for these alterations to the abundances of *Allobaculum*. Starch content was reported as 345 g/kg as fed in the animal protein‐free diet (Bresciani et al., 2018) but was not assessed in the raw‐meat‐based diet (Bermingham et al., 2017). As other studies used overweight dogs (Macedo et al., 2022; Phungviwatnikul et al., 2021) and were not assessed in the meta‐analysis, this hypothesis cannot be effectively challenged in this study. Future investigations could interrogate the effects of dietary fats and starch content on the *Allobaculum* to detail their impacts on the fecal microbiota of the dog.

Consistent with individual studies (Alessandri et al., 2019; Finet et al., 2022; Phungviwatnikul et al., 2021; Xu et al., 2021; You & Kim, 2021), *Fusobacterium*, *Bacteroides*, and *Peptoclostridium* were the dominant genera observed in the fecal microbiome of the dog, though there were differences between dietary protein or fat classifications. The relative abundances of *Alloprevotella, Blautia, Faecalibacterium, Lactobacillus, Megamonas*, and *Prevotella* were also observed to be present in relatively more abundant levels, albeit in a nutrient‐associated manner. Again, these observations were consistent with individual studies included in this meta‐analysis (Finet et al., 2022; Martínez‐López et al., 2021; Phungviwatnikul et al., 2021; You & Kim, 2021).

*Fusobacterium*, despite its association with negative health connotations in humans (Brennan & Garrett, 2019; Lee et al., 2022), is a commensal bacteria genera in the GIT of the healthy domestic dog (Alessandri et al., 2019; Vázquez‐Baeza et al., 2016). *Fusobacterium* is a butyrate producer, utilizing lysine degradation pathways to produce butyrate from protein sources (Louis & Flint, 2017; Vital et al., 2014). This may explain why the relative abundance of *Fusobacterium* increases with increased dietary protein in healthy dogs. Interestingly, the response of *Fusobacterium* to dietary protein was nonlinear, increasing from low to high‐protein and then dropping back down again at supra protein. This suggests that at very high levels of dietary protein, they may be out‐competed by other protein utilisers or that the environmental conditions at supra‐protein levels no longer favor *Fusobacterium*. In terms of its response to dietary fat, the relative abundance of *Fusobacterium* was lowest in the high‐fat classification but increased to approximately 22% of sequence reads in the supra‐fat classification. This may be a confounding effect of the high levels of dietary protein in the supra‐fat diets (39.7% DM).

*Peptoclostridium* also comprises a large component of the commensal population of the fecal microbiome of healthy dogs (Alessandri et al., 2019; Finet et al., 2022; Phungviwatnikul et al., 2021; Xu et al., 2021; You & Kim, 2021). *Peptoclostridium* is also a butyrate producer; however, rather than protein, it instead ferments saccharides such as fructose, glucose, and xylose (Galperin et al., 2016; Pereira et al., 2016) to form butyrate. The relative abundance of *Peptoclostridium* has been shown to decrease in relation to diets containing higher protein content in both healthy and obese dogs (Phungviwatnikul et al., 2021; Xu et al., 2021). In this analysis, the relative abundance of *Peptoclostridium* decreased with higher dietary protein levels consistent with previous results (Phungviwatnikul et al., 2021; Xu et al., 2021). In terms of its response to dietary fat, the relative abundance of *Peptoclostridium* was highest in the high‐fat classification. It is possible that the high‐fat diets also contained significant levels of other macronutrients (e.g., CHOs) to promote *Peptoclostridium* levels.

In terms of its response to dietary protein levels, the relative abundance of *Bacteroides* was highest in the supra‐protein diet classification, whereas in the dietary fat analysis, the relative abundance of *Bacteroides* was lowest in the high‐fat classification. *Bacteroides* are saccharolytic bacteria that are also producers of acetate and propionate (Nogal et al., 2021; Rios‐Covian et al., 2015). *Bacteroides* are found to increase in response to beef‐based protein diets, in comparison to those using chicken as a protein source in dogs (Do et al., 2021; Herstad et al., 2017). However, another study that compared beef‐ and chicken‐based protein diets showed that at the species level, beef protein resulted in increased fecal *Bacteroides vulgatus* and decreased *Bacteroides coprocola* in comparison to dogs fed chicken protein (Beloshapka, 2013).

This is the first attempt, in the canine literature at least, to conduct a meta‐analysis approach to understanding the impacts of diet on the microbiome. This meta‐analysis focussed on the impacts of dietary protein and fat on the fecal microbiome of the domestic dog, due to their nutritional importance to the dog. While the aim of this meta‐analysis explicitly focussed on the impacts of dietary protein and fat on the fecal microbiome of the domestic dog, we recognize the limitation of not including other dietary components, specifically those known to be major drivers of the fecal microbiome such as dietary fiber (Beloshapka, 2013). While the concepts of a meta‐analysis have largely been followed in the current study, especially in terms of PRISMA guidelines (Moher et al., 2009), there are a number of factors that limit the statistical power of this analysis. This includes missing data from approximately 40‐odd publications that did not have or were not able to provide, publicly available data sets (Supplementary Data 1 for DOI: 10.1002/mbo3.1404 (figshare.com)). An additional 11 studies could not be included in the meta‐analysis as they did not publish complete diet information. Similarly, we were unable to examine the impacts of explanatory factors such as age, breed, and neuter status as not all publications included these explanatory data. A limitation of the findings in this study is that there may be confounding impacts of dietary classifications as a result of using published literature. For example, diets classed as high protein may also have had corresponding high‐fat levels (Kilburn et al., 2020; Moinard et al., 2020; Sandri et al., 2020; Schauf et al., 2018). It is possible that, with the ability to increase the number of publications included in future meta‐analyses, these confounding issues could be addressed and minimized. Other limitations include the inconsistency in sample handling, DNA extraction, primer sets, and sequencing methodologies in these studies. Most studies used the V3–V4 16S primer set to measure the prokaryotic taxa but four did not, two of those being WGS, which also introduced biases in the proportion of taxa (Tremblay et al., 2015). As WGS becomes more common in metagenomic studies, there is a question of how to enumerate taxa from the sequencing reads, particularly around which database (Khachatryan et al., 2020). To strengthen the scientific community's ability to further our knowledge of GIT interactions in dogs (and cats) and as noted by other authors (e.g., Bisanz et al., 2019), there should be improvements and standardization of reporting of explanatory factors (including dietary nutrients) in studies investigating the impacts of diet on microbial composition accountability. Finally, the genera associated with separation between dietary treatment groups were largely present in relatively low numbers. Ideally, the levels and identity of these bacteria would be verified using techniques such as quantitative polymerase chain reaction. This is because the identification of low‐abundant taxa will depend on sequencing depth, and if this is insufficient, many of these taxa will remain undetected (Sung et al., 2023). However, this validation is not possible when using meta‐analysis approaches. Nonetheless, this meta‐analysis has demonstrated the opportunities for a more in‐depth analysis of the impacts of diet on the fecal microbiome by using the existing literature to highlight overlooked or unnoticed trends.

## 5 CONCLUSION

This meta‐analysis has suggested that changes to the commensal bacteria that are present in relatively low abundances may provide key insights into the impacts of diet on the fecal microbiome of the dog. Future research would benefit from investigating the t role of low‐abundant genera such as *Sharpea, Clostridium senso stricto 13*, and *Prevotellaceae* Ga6A1 group in terms of their role in the GIT function of the domestic dog.

## AUTHOR CONTRIBUTIONS

**Francis D. Phimister**: Conceptualization; investigation; writing—original draft; writing—review and editing; methodology; formal analysis. **Rachel C. Anderson**: Conceptualization; funding acquisition; methodology; writing—review and editing; writing—original draft; supervision. **David G. Thomas**: Conceptualization; writing— original draft; writing—review and editing; supervision. **Michelle J. Farquhar**: Conceptualization; investigation; funding acquisition; writing—original draft; writing—review and editing; methodology; supervision. **Paul Maclean**: Writing— original draft; writing—review and editing; methodology; formal analysis; visualization; software. **Ruy Jauregui**: Writing—original draft; formal analysis. **Wayne Young**: Writing—original draft; writing—review and editing; formal analysis; methodology. **Christina F. Butowski**: Methodology; writing—review and editing; writing—original draft; formal analysis. **Emma N. Bermingham**: Conceptualization; investigation; funding acquisition; writing—original draft; writing—review and editing; methodology; supervision.

## CONFLICT OF INTEREST STATEMENT

Emma N. Bermingham, Rachel C. Anderson, Christina F. Butowski, Wayne Young, and Paul Maclean were employed by AgResearch. David G. Thomas was employed by Massey University. Michelle J. Farquhar was employed by Mars Petcare. Emma N. Bermingham has received funding from Mars Petcare.

## ETHICS STATEMENT

None required.

## Data Availability

This publication is supported by multiple data sets, which are openly available at publication locations cited in Table A2.

## 1

**Table A1:** Region of 16S covered by the primer set in each study.

Study	V1	V2	V3	V4	V5	V6
ID9						
ID18						
ID19						
ID23						
ID24						
ID25						
ID27						
ID28						
ID29	Whole‐genome shotgun sequencing					
ID30						
ID32						
ID38						
ID39						
ID43						
ID44						
ID45	Whole‐genome shotgun sequencing					

**Table A2:** Meta‐data of publications used to assess the effects of dietary crude protein (protein) and/or crude fat (fat) on the composition of the fecal microbiome in the domestic dog.

Study ID	Authors	Reference	DOI	Overall number of dogs	Diet ID	Reported protein content (% DM)	Protein treatment	Reported fat (% DM)	Fat treatment	Number of dogs on a diet
ID9	Bermingham et al.^a^	Bermingham et al. (unpublished)		15	Diet 1	39.1	High^b^	19.8	Moderate^c^	8
					Diet 2	24.7	Moderate	12.3	Low	7
ID18	Scarsella et. al.	Scarsella et al. (2020)	https://doi.org/10.3390/ani10030531	8	Diet 1	29.1	Moderate	17.3	Moderate	8
ID19	Sandri et al.	Sandri et al. (2020)	https://doi.org/10.1016/j.aninu.2020.03.003	27	Diet 1	29.7	Moderate	21.7	High	9
					Diet 2	28.9	Moderate	21.7	High	9
					Diet 3	30.1	High	22.8	High	9
ID23	Kirchoff et al.	Kirchoff et al. (2019)	https://doi.org/10.7717/peerj.6103	31	Diet 1	27.8	Moderate	15.6	Moderate	30
					Diet 2	32.2	High	19.4	Moderate	1
ID24	Pilla et al.	Pilla et al. (2019)	https://doi.org/10.3389/fvets.2019.00277	8	Diet 1	22.7	Low	10.8	Low	8
ID25	Sandri et al.	Sandri et al. (2019)	https://doi.org/10.1080/1828051X.2019.1645624	8	Diet 1	27.2	Moderate	19.2	Moderate	8
					Diet 2	26.0	Moderate	19.0	Moderate	8
					Diet 3	23.9	Low	15.2	Moderate	8
ID27	Jarett et al.	Jarett et al. (2019)	https://doi.org/10.7717/peerj.7661	32	Diet 1	26.2	Moderate	14.2	Low	8
					Diet 2	27.4	Moderate	16.0	Moderate	8
					Diet 3	26.7	Moderate	14.7	Low	8
					Diet 4	25.8	Moderate	14.1	Low	8
ID28	Bresciani et al.	Bresciani et al. (2018)	https://doi.org/10.1111/jvim.15a227	14	Diet 1	22.0	Low	13.6	Low	14
ID29	Coelho et al.	Coelho et al. (2018)	https://doi.org/10.1186/s40168-018-0450-3	32	Diet 1	27.3	Moderate	17.5	Moderate	16
					Diet 2	53.9	Supra	18.4	Moderate	16
					Diet 3	30.0	Moderate	18.2	Moderate	32
ID30	Schauf et al.	Schauf et al. (2018)	https://doi.org/10.1093/jas/sky264	12	Diet 1	28.5	Moderate	10.9	Low	12
					Diet 2	32.5	High	23.2	High	12
ID32	Li et al.	Li et al. (2017)	https://doi.org/10.1128/mbio.01703-16	32	Diet 1	53.3	Supra	15.1	Moderate	15
					Diet 2	27.6	Moderate	15.7	Moderate	16
					Diet 3	30.5	High	17.2	High	16
ID38	Kilburn et al.	Kilburn et al. (2020)	https://doi.org/10.3389/fmicb.2020.564160	8	Diet 1	46.9	Supra	32.1	Supra	8
					Diet 2	42.7	High	37.2	Supra	8
					Diet 3	40.0	High	41.9	Supra	8
					Diet 4	38.2	High	46.5	Supra	8
ID39	Beloshapka et al.	Beloshapka et al. (2021)	https://doi.org/10.1093/jas/skaa409	7	Diet 1	37.7	High	13.2	Low	7
ID43	Moinard et al.	Moinard et al. (2020)	https://doi.org/10.3389/fvets.2020.566282	24	Diet 1	30.6	High	34.5	Supra	24
ID44	Martínez‐López et al.	Martínez‐López et al. (2021)	https://doi.org/10.1186/s42523-021-00101-8	46	Diet 1	21.1	Low	6.6	Low	46
					Diet 2	19.0	Low	14.4	Low	46
					Diet 3	19.2	Low	8.7	Low	46
ID45	Eisenhauer et al.	Eisenhauer et al. (2019)	https://doi.org/10.1093/jas/skz264	10	Diet 1	21.3	Low	10.3	Low	10
					Diet 2	40.9	High	10.9	Low	10
					Diet 3	21.6	Moderate	10.1	Low	10
					Diet 4	39.9	High	10.5	Low	10
					Diet 5	21.9	Moderate	9.9	Low	10

*Note*: Diet ID indicates the number of diets used within each study; treatment refers to the classification of the % dry matter (DM) protein or fat content in the diet (low, moderate, high, or supra).

^a^ Manuscript in preparation. Data are available from the author on request.

^b^ Low protein was classed as <25% crude protein content by dry matter (% DM) analysis. Moderate protein was between 25% and 30% DM. High protein was between 30% and 45% DM. Any reported crude protein content higher than 45% DM was classed as supra protein.

^c^ Low fat was classed as <15% DM dietary fat content. Moderate fat was between 15% and 20% DM. High fat was between 20% and 30% DM. Any reported dietary fat content higher than 30% DM was classed as supra fat.

See Tables A1, A2, and Figures A1, A2, A3, A4.
